# Near-Infrared Fluorescence of Silicon Phthalocyanine Carboxylate Esters

**DOI:** 10.1038/s41598-017-12374-8

**Published:** 2017-09-25

**Authors:** Amlan K. Pal, Shinto Varghese, David B. Cordes, Alexandra M. Z. Slawin, Ifor D. W. Samuel, Eli Zysman-Colman

**Affiliations:** 1 Organic Semiconductor Centre, EaStCHEM School of Chemistry, University of St Andrews, St Andrews, Fife, KY16 9ST UK; 2 Organic Semiconductor Centre, SUPA School of Physics and Astronomy, University of St Andrews, St Andrews, Fife, KY16 9SS UK; 3 EaStCHEM School of Chemistry, University of St. Andrews, St. Andrews, KY16 9ST Fife, United Kingdom

## Abstract

Seven silicon(IV) phthalocyanine carboxylate esters (SiPcs, **1**–**7**) with non-, partially- and per-fluorinated aliphatic (linear or branched at the alpha-carbon) and aromatic ester groups have been synthesized, their solid-state structures determined and their optoelectronic properties characterized. The SiPcs exhibit quasi-reversible oxidation waves (*vs*. Fc^+^/Fc) at 0.58–0.75 V and reduction waves at −0.97 to −1.16 V centered on the phthalocyanine ring with a narrow redox gap range of 1.70–1.75 V. Strong absorbance in the near-infrared (NIR) region is observed for **1**–**7** with the lowest-energy absorption maximum (Q band) varying little as a function of ester between 682 and 691 nm. SiPcs **1**–**7** fluorescence in the near-infrared with emission maxima at 691–700 nm. The photoluminescence quantum yields range from 40 to 52%. As a function of esterification, the SiPcs **1**–**7** exhibit moderate-to-good solubility in chlorinated solvents, such as 1,2-dichlorobenzene and chloroform.

## Introduction

Phthalocyanines (Pcs) are one of the most extensively studied classes of organic functional macrocycles, having four nitrogen-bridged isoindole units^1,2^. Pcs are known for their robust thermal and chemical properties, and their diverse optoelectronic and magnetic properties, especially their strong absorption in the near Infrared (λ_abs_ ~ 700 nm). These attractive optoelectronic properties have catalysed the use of Pcs as dyes and pigments^3^, and as components in electrochromic^4^ and optical limiting devices^5,6^, organic thin-film transistors^7,8^, organic light emitting diodes (OLEDs)^9^, multistage-redox-dependent fluorophores^10^, organic (OSC)^11,12^ and dye-sensitized solar cells (DSSCs)^13,14^, and also in NIR fluorescence imaging^12,15,16^.

Many of the solid-state properties of Pcs are highly dependent on the extent of intermolecular π-π stacking interactions, especially in aggregates in polar protic solvents such as water or ethanol^17^. Pcs are normally characterised by an intense absorption of the Q-bands in the near infrared (NIR) region of ~700 nm^18^. Dimerization or aggregation between neighbouring Pc molecules generally leads to fluorescence quenching and a broadened, red-shifted principal Q-band due to electronic/exciton coupling interactions^19,20^ that can normally be observed even in very dilute solutions. Aggregation is an unfavorable property of Pcs since it can not only cause difficulties in purification and characterization^21^ but also result in the quenching of luminescence due to enhanced non-radiative excited state decay^22^, and therefore lowers the photoluminescence quantum yields, Φ_PL_. In the context of achieving non-aggregated Pc derivatives, several approaches have been adopted. These include introduction of bulky peripheral substituents such as *tert*-butyl groups^14,23,24^ and rigid spirocyclic fused rings^25^, and the introduction of metal ions such as Sn(IV) and Ln(III) to break the planarity of the Pc-containing compounds^26,27^ such that the metal ion sits outside the plane of the Pc macrocycle. Another viable approach is to introduce elements such as Ru(II), Si(IV) or Ge(IV), that possess more than four valencies. Many Pc derivatives have been explored including main group metals and 1^st^–3^rd^ row transition metals such as Al(III), Cu(II), Ni(II), Co(II), Zn(II), Ga (III), In(III), Sn(IV), Ge(IV), Ln(III), Pt(II) exhibiting square-planar, square-pyramidal, or octahedral coordination geometries^18^. Pcs containing Si(IV) are an attractive subclass of Pcs because of the elemental abundance and very low toxicity levels of silicon coupled with their low band gap (~1.7 eV). The SiPcs with axial oxygen-terminated ligands (alkoxide, siloxide, aryloxide, and carboxylate) have been known since the 1960s^28,29^ and they have been applied as photosensitizing agents in photodynamic therapy^30^. Recently, SiPcs with siloxide and aryloxide ligands have found use as chromophores and fluorophores in DSSCs and OLEDs^31^, the latter with external quantum efficiencies of up to 4.5%^13^. In contrast, to the best of our knowledge, SiPcs with carboxylate ligands have rarely been used in organic electronics. Considering the strong nature of Si-O bond, the wide range of commercially-available carboxylic acids and their capacity to confer increased solubility, silicon phthalocyanine carboxylates are an attractive target for the design of solution-processable electron-accepting materials for organic electronics.

**Figure 1:**
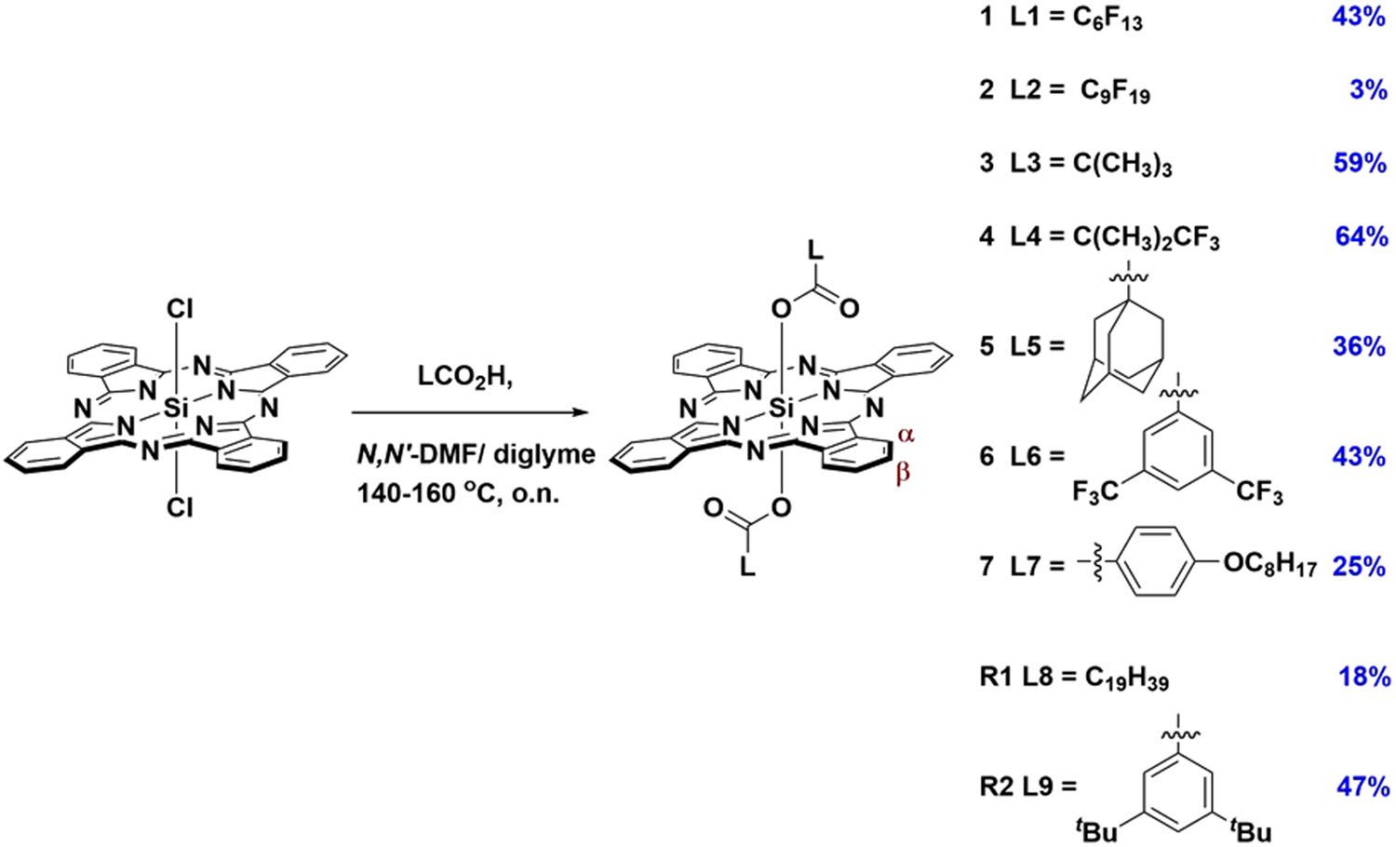
Synthesis of SiPc complexes **1**–**7** and benchmark complexes **R1** and **R2**. Proton labelling scheme (α and β protons) for the macrocycle ring is also shown.

In a recent study, we investigated the optoelectronic properties of two SiPcs bearing axial alkyl/aryl-carboxylate ligands (**R1** and **R2**, Fig. 1) and explored their use in OLED and OSC devices^32^. Herein, we report a systematic study of the synthesis and optoelectronic characterization of a series of axially substituted SiPc carboxylates, **1**–**7**, bearing perfluorinated (**1** and **2**) and partially fluorinated (**4**) carboxylates, alkyl carboxylates (**3** and **5**) and aryl carboxylates (**6** and **7**) (Fig. 1). The new SiPcs were comprehensively characterized by ^1^H and ^19^F NMR spectroscopy, NSI^+^ high-resolution or MALDI mass spectrometry, melting point determination, elemental analysis and single crystal XRD. The effect of different axial substituents on the optoelectronic properties of these complexes is discussed, supported by Density Functional Theory (DFT) calculations, with the results compared to our previously reported reference compounds **R1** and **R2**.

SiPcs **1**–**7** were synthesized by nucleophilic substitution reactions of commercially available silicon phthalocyanine dichloride, SiPcCl_2_ with an excess of the corresponding carboxylic acids, either in DMF (SiPc **3**) or in diglyme (SiPcs **1**, **2**, **4**–**7**) at 140–165 °C (Fig. 1) in poor to good yields (3–64%) based on the carboxylic acids. The SiPcs were purified by column chromatography on silica. The product typically elutes as the first blue band from the column and it is usually well separated from the other fractions. Compounds **1**–**7** were obtained as air and moisture stable blue solids. It was found that the choice of solvent plays a crucial role in the synthesis of the SiPcs. For instance, **1** and **6** can be prepared from diglyme, but not from DMF. The aliphatic carboxylates were either linear at the alpha-carbon (**1**–**2**) or branched (**3**–**5**). The aromatic carboxylates possessed solubilizing groups (**6** and **7**) that were either electron-withdrawing (trilfuoromethyl in **6**) or electron-donating (octyloxy in **7**).

All the compound characterization data are consistent with the proposed SiPc structures and further confirmed by single crystal data. Compounds **2**–**7** show melting points >300 °C while **1**, possessing a long-chain aliphatic carboxylate C_19_H_39_CO_2_^−^ ligands shows a significantly lower melting point of 168–170 °C. Fluorinated SiPcs **1**, **4**, and **6** exhibit low solubility of <5 mg mL^−1^ in chlorinated solvents, such as 1,2-dichlorobenzene and chloroform while the other SiPcs are soluble in those solvents (>10–30 mg mL^−1^) but are insoluble in acetonitrile and alkanes. The solubility of SiPcs **2**, **3**, **5** and **7** in 1,2-dichlorobenzene are comparable to the same reported for reference complexes **R1** (>10 mg mL^−1^) and **R2** (>35 mg mL^−1^) in 1,2-dichlorobenzene^32^.

**Figure 2:**
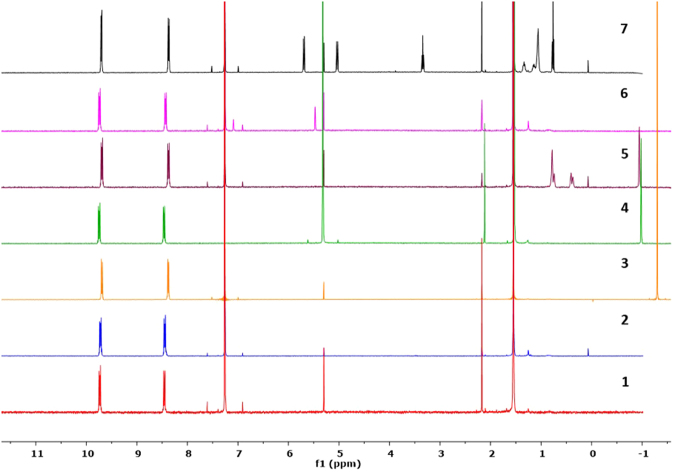
Stacked ^1^H NMR spectra of **1**–**7** in CDCl_3_ at 400 MHz at r.t. (the ^1^H NMR spectrum of **4** was recorded in CD_2_Cl_2_ at 400 MHz at r.t.).

The axially-substituted SiPcs have characteristic ^1^H NMR spectra (Fig. 2). The ^1^H resonances of the axial substituents close to the phthalocyanine ring are strongly shifted upfield as they experience a large diamagnetic ring-current shielding effect induced by the macrocyclic ring. This effect is more pronounced in **3**–**5** where some of the aliphatic protons appear ≤ −1 ppm. The phthalocyanine ring ^1^H resonances appear as two downfield-shifted unresolved multiplets integrating each as 8 H at around 9.8 and 8.5 ppm, for the α and β protons, respectively^26^. The two axially substituted carboxylate ligands are symmetric on the NMR timescale and exhibit a single set of NMR resonances. This observation in conjunction with the presence of the two sets of phthalocyanine ring ^1^H resonances suggest a local *D*_*4h*_ symmetry in **1**–**7** in solution state, irrespective of ester ligand. Electrospray ionization mass spectrometry showed the expected isotope distribution pattern for both the [M + H]^+^ and [M + Na]^+^ ions for **1**–**7**.

## Crystal structures

The crystal structures of **1**–**7** were determined. Details of structure refinements and some crystallographic parameters are collected in Supplementary Table S1 in the ESI. Selected bond distances of SiPcs **1**–**7**, **R1** and **R2** are gathered in Supplementary Table S2. The structure of **6** shows two independent molecules in the asymmetric unit, as well as a co-crystallized molecule of acetonitrile. The flexible polyfluorinated chains of the carboxylate ligand in **2** result in a lower quality, but unambiguous, determination of its structure.

**Figure 3:**
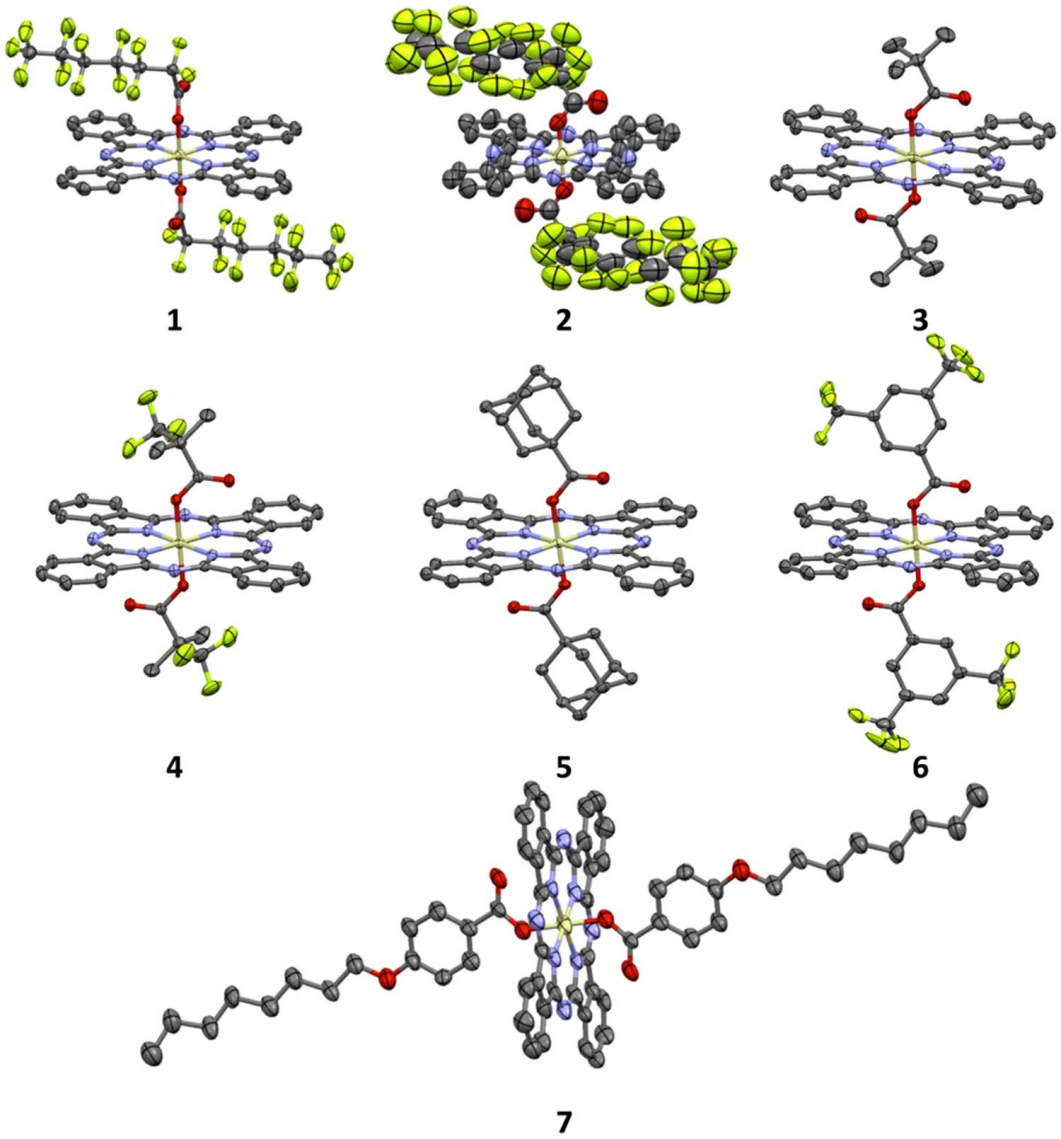
X-ray structures of **1**–**7**, with ellipsoids drawn at the 50% probability level. Hydrogen atoms, solvent molecules, minor disorder components, and additional independent molecules are omitted for clarity.

In all seven structures, the hypervalent silicon(IV) adopts the expected distorted octahedral SiN_4_O_2_ coordination environment (Fig. 3)^32^. Except in the case of **6**, the SiPc has a crystallographic inversion center at the silicon atom; therefore, the SiN_4_ fragment is planar, irrespective of any distortions within the phthalocyanine ring itself, the SiO_2_ fragment is linear, and only two of the Si–N and one of the Si–O bonds are unique. In **6**, the same planar SiN_4_ fragment (root-mean-squared deviation of the SiN_4_ fragment from planarity, 0.0019 and 0.0003 Å, for Si1 and Si51, respectively), and linear SiO_2_ fragment [O–Si–O angles, 179.59(9) and 179.40(8)°, for Si1 and Si51, respectively] are seen, despite the lack of a center of symmetry at Si. The Si–N bonds are significantly longer [1.8897(18)–1.9203(11) Å] than the Si–O bonds [1.7358(9)–1.7793(15) Å]. The Si–N bond lengths decrease while the Si–O bond lengths increase when the carboxylate ester becomes more electron-withdrawing from a non-fluorinated to a (per-)fluorinated one (Supplementary Table S2). All Si–N and Si–O bond distances fell within the range of distances for six-coordinate SiPc compounds with axial O-donor ligands, found in a search of the Cambridge Structural Database (CSD version 5.37)^33,34^. The Si–N distances were seen to lie below the middle of the range of distances (1.89–1.95 Å), whereas the Si–O distances were at the upper end of the range (1.65–1.78 Å).

**Figure 4:**
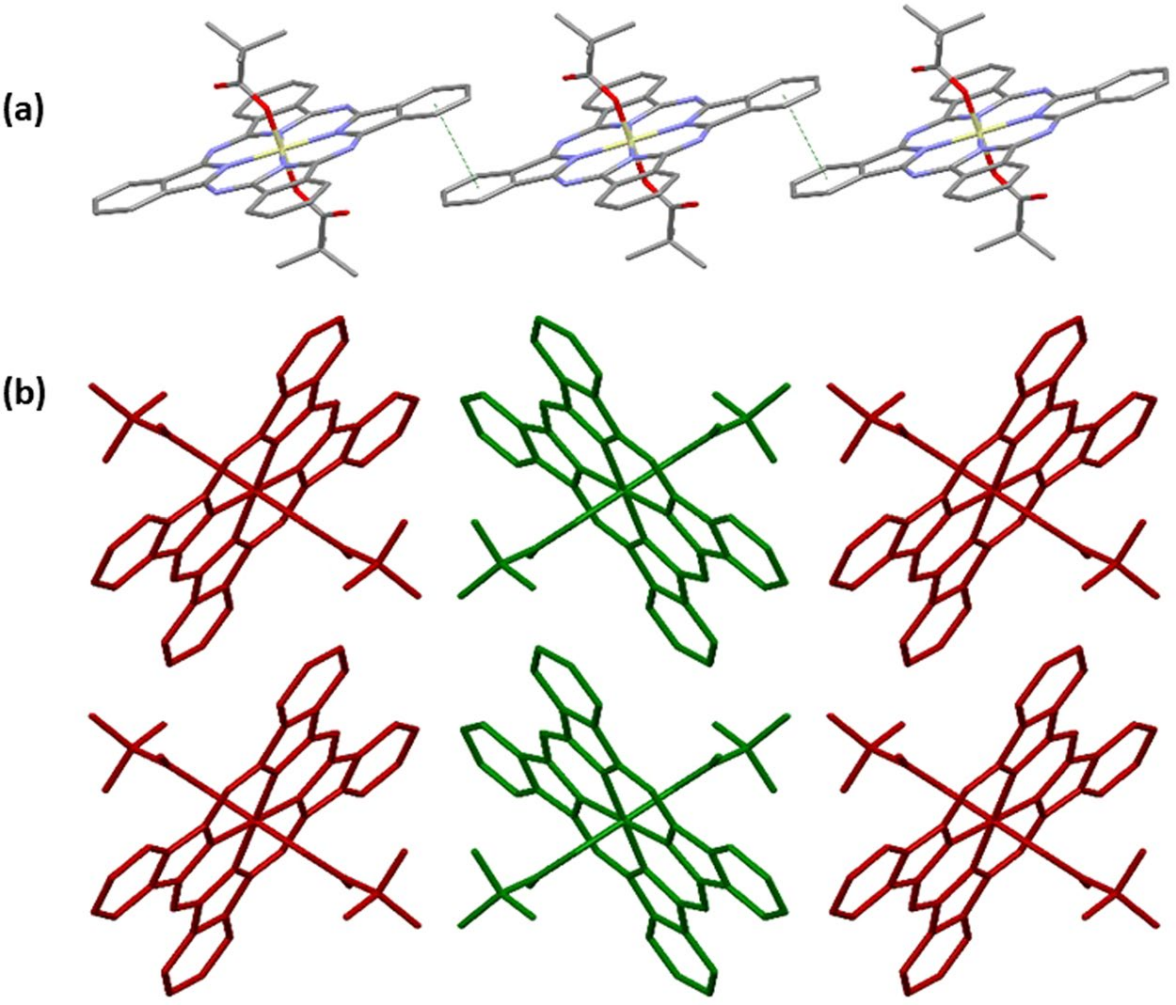
Views of sections of the structure of **3** showing (**a**) the single-chain mode of π···π interactions and (**b**) the resulting herringbone packing motif. Hydrogen atoms are omitted for clarity.

**Figure 5:**
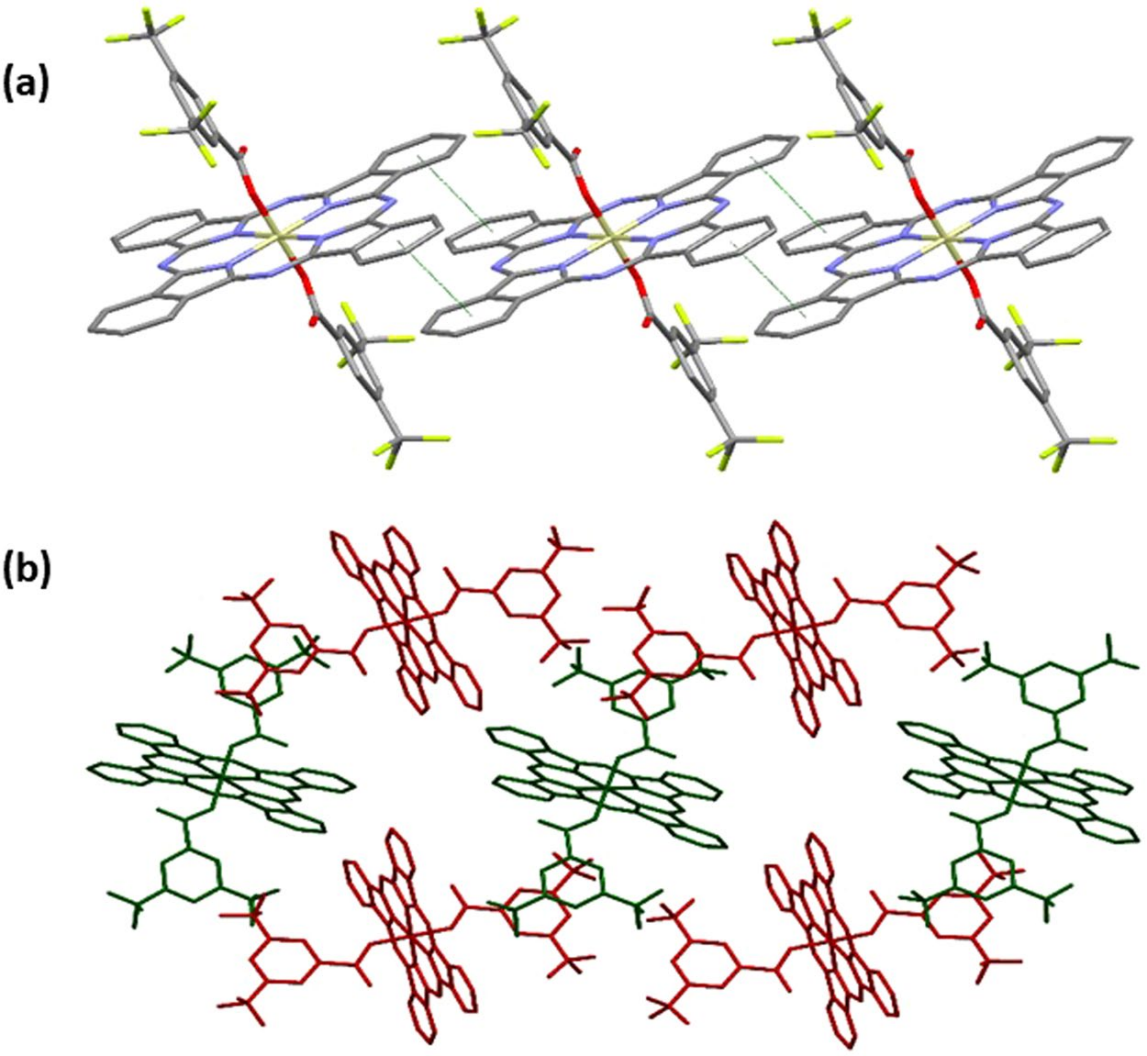
Views of sections of the structure of **6** showing (**a**) the double-chain mode of π···π interactions and (**b**) the herringbone packing motif that results. Hydrogens Hydrogen atoms are omitted for clarity.

The complexes show a limited selection of both π···π and C–H···π interactions; however, these do not involve the aryl groups of the carboxylate ligands in **6** and **7**, which is likely due to the surrounding steric bulk. In contrast, the phenyl rings of the phthalocyanines do participate in π–stacking, with centroid···centroid distances ranging from 3.5361(11) to 3.7795(19) Å. The exceptions to this occur in **1** and **5**, which show no centroid···centroid distances less than 3.8 Å, although there are phenyl rings positioned appropriately at distances slightly greater than this that show patterns of interactions in line with the other structures. This π–π stacking occurs through either the two *trans*-benzenes to give an infinite single chain (**3** and **4**, Fig. 4) or through the two pairs of *cis*-benzenes to give an infinite double-chain (**1**, **2**, **5**, **6**, and **7**, Fig. 5). As well as these π···π interactions, compounds **5** and **7** also show weak C–H···π interactions between aliphatic C–H groups in the axial ligands and aromatic rings in either the phthalocyanine, or the axial ligand, at distances of 3.00 and 2.71 Å, respectively. The interaction in **5** is at the conventional van der Waals limit, but C–H···π interactions have been suggested to be effective at distances beyond this value^35^.

All the complexes, apart from **5**, exhibit weak intermolecular C–H···O hydrogen bonds to the carbonyl oxygen of the carboxylate ligand, which acts as acceptor for two C–H···O interactions simultaneously. These predominantly involve aromatic phthalocyanine C–H groups, but also aliphatic C–H groups in some cases. The H···O separations vary between 2.37 and 2.55 Å, with corresponding C···O separations of 3.265(3) to 3.445(13) Å. For **5**, the steric bulk of the adamantyl group may restrict some close-packing interactions with the adjacent carboxylates. The SiPcs with fluorinated carboxylate ligands (**1**, **2**, **4**, and **6**) additionally exhibit weak intermolecular C–H···F interactions. These show a similar distance spread to the C–H···O hydrogen bonds, with H···F separations varying between 2.36 and 2.62 Å, and C···F separations between 3.169(4) to 3445(3) Å. Such interactions would generally be considered too weak to make a significant contribution to the packing, except that they are seen to occur in cooperative pairs; working in conjunction with other C–H···F interactions. The exception to this pattern is in **4**, where no other interactions are seen to be operating cooperatively with the C–H···F interactions, suggesting that in this case the close H···F contact may just be an artifact of packing.

**Figure 6:**
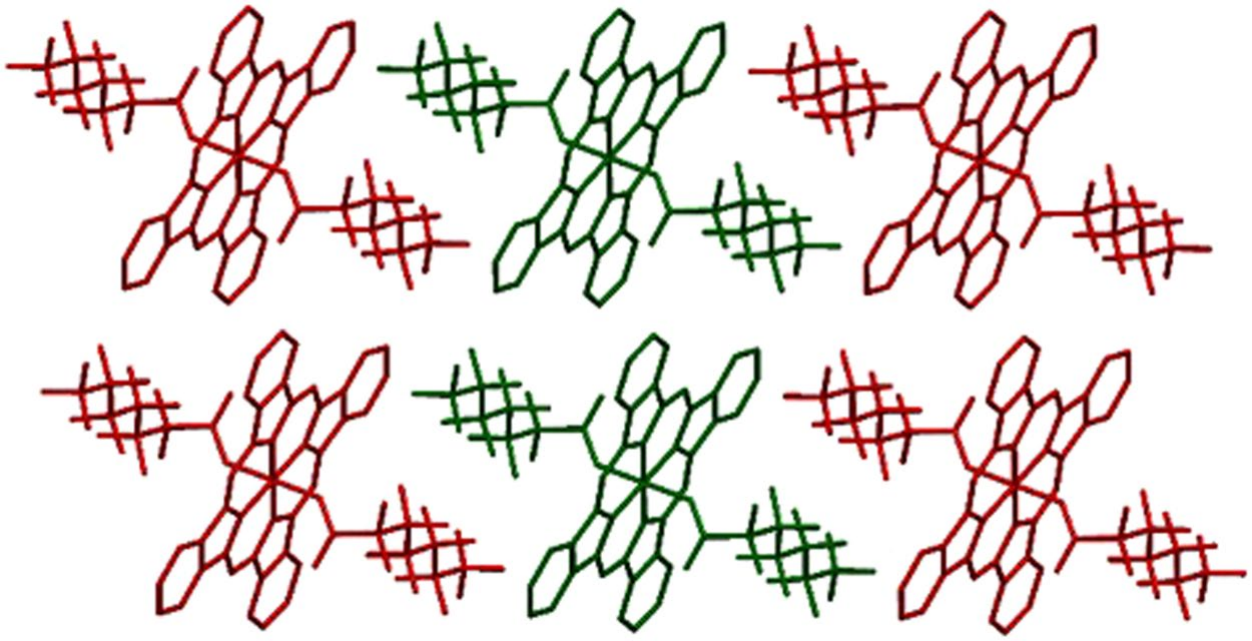
View of a section of the structure of **1** showing the all-parallel packing motif. Hydrogen atoms are omitted for clarity.

The presence of the axial ligands can lead to disruption of intermolecular interaction in the solid state. The linear alkyl chains of the carboxylates in **1** and **2** are arranged parallel to the plane of the phthalocyanine ring (Fig. 6), whereas all the other carboxylate ligands are arranged nearly orthogonal to it, even in the case of **7**, containing an aryl octyl ether (Fig. 3). This arrangement of axial ligands is mirrored in other SiPcs, where the presence of aryl carboxylate ligands show positioning orthogonal to the SiPc^32,36,37^, while alkyl carboxylates show a parallel position^26,32,37–40^. Potentially arising from these differing chain orientations, and the resulting presence or absence of steric bulk across the face of the SiPc, the phthalocyanine rings pack with the ring-planes arranged in a parallel fashion in **1**, **2** and **5**, whereas in the rest of the SiPcs they pack in a herringbone pattern, with SiPc planes approximately orthogonal. This pattern of arrangements is not mirrored as closely in other known structures; although those with aryl carboxylates do show packing with orthogonal SiPcs^37^, those with alkyl carboxylates show a mixture of packing motifs, most showing a parallel arrangement of SiPcs^37,39^, but some showing herringbone packing^37,40^, or other motifs with orthogonally arranged SiPcs^36,39^. The intermolecular separation was estimated using the shortest Si···Si distance in each structure. Surprisingly, the longest separation is found to be in **4** (Table 1), which has the highest solubility in the series, and the shortest in **7**.

**Table 1:** Redox data^*a*^ of complexes **1**, **3**–**7** (in degassed MeCN) and some benchmark complexes.

Cmpd	*E*_1/2_^ox^ (ΔEp)	*E*_1/2_^red^ (ΔEp)	ΔE_redox_^b^	E_HOMO_^c^	E_LUMO_^c^	|Ε_LUMO-HOMO_|^c^
1	0.75 (60)	−0.97 (59)	1.72	−5.40	−3.33	2.07
3	0.60 (96)	−1.15 (96), −1.61 (97)	1.75	−5.27	−3.17	2.10
4	0.63 (60)	−1.09 (60), −1.54 (71)	1.72	−5.30	−3.21	2.09
5	0.58 (83)	−1.16 (96), −1.63 (85)	1.74	−5.09	−2.97	2.12
6	0.70 (71)	−1.03 (61), −1.47 (61)	1.73	−5.35	−3.27	2.08
7	0.58 (60)	−1.11 (60), −1.52 (59)	1.69	−5.25	−3.15	2.10
R1	0.59	−1.16	1.75	−5.12	−3.00	2.12
R2	0.60	−1.14, −1.57	1.74	−5.07	−2.95	2.12

^a^Potentials are in volts (V) *vs*. Fc/Fc^+^ in dichloromethane solutions, 0.1–0.2 M in [*n*-Bu_4_N]PF_6_, recorded at room temperature at a sweep rate of 100 mV/s using a glassy carbon electrode as a working electrode, a platinum-spiral counter electrode and a platinum wire as a reference electrode. The difference between cathodic, E_pc_, and anodic, E_pa_, peak potentials, ΔE_p_, (millivolts) is given in parentheses. ^b^ΔE_redox_ is the difference (V) between first oxidation and first reduction potentials. ^c^DFT calculated energy in eV.

### Electrochemistry

**Figure 7:**
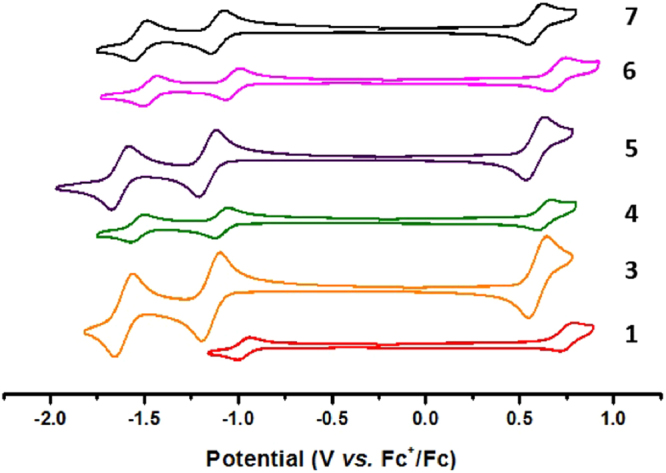
Cyclic voltammograms of **1** and **3**–**7** in degassed dichloromethane, recorded at a scan rate of 100 mV/s.

**Figure 8:**
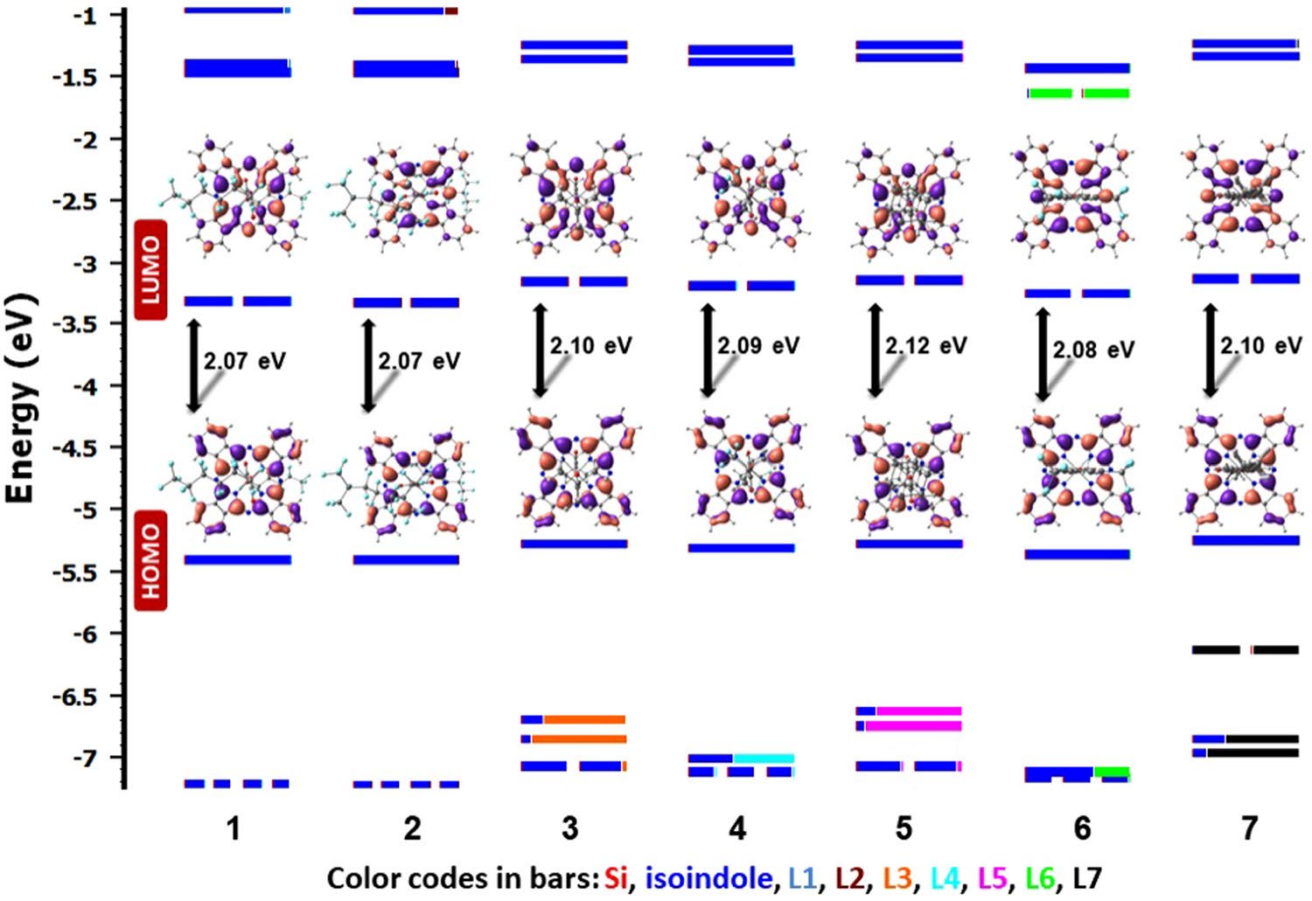
Calculated frontier MO energies of **1**–**7**, obtained from DFT [(B3LYP/SBKJC-VDZ for Si(IV)) and (6–31 G** for C,H,N,O,(F)] with CPCM(MeCN) and 0.5 eV threshold of degeneracy (orbitals are isocontoured at 0.03). Kohn-Sham MOs of **1**–**7** are also shown.

The cyclic voltammograms of compounds **1** and **3**–**7** were obtained in dichloromethane at room temperature and are shown in Fig. 7 and the electrochemical data collated in Table 1. Due to poor yield, the electrochemical measurement of **2** was not possible. At positive potential, the SiPcs exhibit mono-electronic quasi-reversible oxidations in the range 0.58–0.75 V *vs*. Fc/Fc^+^. DFT calculations indicate that the highest occupied molecular orbitals (HOMOs) are distributed on the isoindole moieties of the macrocycles (Fig. 8). Based on the DFT calculations, and in line with the literature data, the first oxidation processes of **1** and **3**–**7** are assigned to the removal of one electron from the isoindole moiety^31^. The lower energies calculated for the HOMOs of **1** (E_HOMO_ = −5.40 eV), **3** (E_HOMO_ = −5.27 eV) and **4** (E_HOMO_ = −5.30 eV) compared to that of **R1** (E_HOMO_ = −5.12 eV) are in good agreement with their more positive anodic potentials (Table 1). The slightly shallower HOMO of **5** (E_HOMO_ = −5.09 eV) bearing the adamantyl moiety compared to that of **R1** (E_HOMO_ = −5.12 eV) is in line with the 10 mV cathodic shift of its oxidation potential compared to that of **R1**. The energy calculated for the HOMO (E_HOMO_ = −5.35 eV) of bis(trifluoromethyl)phenyl carboxylate-substituted compound **6** is significantly lower than that of **R2** (E_HOMO_ = −5.07 eV), aligning with the 100 mV anodic shift of the oxidation potential of **6** compared to that of **R2**. However, the trend observed in the lower HOMO energy calculated for **7** (E_HOMO_ = −5.25 eV) compared to that of **R2** (E_HOMO_ = −5.07 eV) is not in line with the observed 200 mV cathodic shift in its oxidation potential compared to that of **R2**. So, in general, when electron-withdrawing fluorinated groups are added to the carboxylate ligand, the redox potentials are shifted positively, the oxidation becomes harder; e.g., compare (**1** and **4**) *vs*. (**3** and **5**) or **6** *vs*. **7** (Table 2). A similar anodic shift of 30 mV is also observed on substitution of one methyl group in the *t*Bu group in **3** with a -CF_3_ group in **4**. Compared to **R1**, all the fluorinated SiPcs, **1**, **4**, and **6** exhibit anodically shifted oxidation potentials as a function of incorporation of electron-withdrawing -CF_3_ groups (Table 1). The similar argument is also valid for the observed 100 mV anodic shift in the oxidation potential of SiPC **6** compared to that of **R2** (Table 1). The most positive oxidation potential in the series is observed for **1**, which contains the perfluoroheptanoate axial ligands.

**Table 2:** UV-vis absorption data in dichloromethane at room temperature.

SiPc	*λ*_abs_/nm (*ε*/10^4^ M^−1^ cm^−1^)
1	295 (2.8), 335 (6.9, sh), 356 (7.8), 621 (3.8), 659 (3.3), 691 (29)
2	295 (2.8), 335 (6.8, sh), 355 (7.6), 621 (3.7), 659 (3.2), 690 (27)
3	290 (2.6), 358 (7.2), 613 (3.9), 651 (3.3), 682 (27)
4	293 (2.3), 358 (7.4), 616 (3.7), 654 (3.1), 685 (26)
5	290 (2.4), 335 (5.8, sh), 350 (6.2, sh), 360 (6.5), 614 (3.7), 652 (3.1), 683 (26)
6	295 (2.4), 358 (7.8), 618 (3.7), 657 (3.2), 688 (27)
7	257 (6.1), 291 (2.3), 359 (7.2), 615 (3.8), 653 (3.2), 684 (27)

Upon scanning to negative potentials, the SiPcs exhibit one or two quasi-reversible monoelectronic reductions, the first at −0.97 to −1.16 V and the second at −1.47 to −1.63 V, which are typical of Pcs^41^. For all the SiPcs, DFT calculations point to a lowest unoccupied molecular orbital (LUMO) that has isoindole character coupled with the contribution from the bridging N-atoms. Therefore, and in line with the literature data, the first reduction can be assigned to reduction of the macrocycle itself. Similar to the trend observed in the oxidation of (**1** and **4**) *vs*. (**3** and **5**), the presence of the electron-withdrawing fluorinated groups anodically shifts the first reduction potential of (**1** and **4**) by 60–190 mV compared to those of (**3** and **5**) (Table 2). This trend is in good agreement with the more stabilized computed LUMOs of **1** and **4** (E_LUMO_ = −3.33 eV for **1** and −3.21 eV for **4**) compared to those of **3** and **5** (E_LUMO_ = −3.17 eV for **3** and −2.97 eV for **5**). The computed and experimentally determined LUMO energies of the aryl-substituted SiPcs **6** and **7** containing either electron-withdrawing -CF_3_ or electron-donating octyloxy groups, respectively, also follow the expected trend. The first reduction potential of **6** is 80 mV anodically shifted compared to that of **7**; the LUMO of **6** (E_LUMO_ = −3.27 eV for **6**) is likewise more stabilised compared to the LUMO of **7** (E_LUMO_ = −3.15 eV for **7**) (Table 2). DFT calculations demonstrate that the LUMO + 1 is centred on the isoindole moiety in all the compounds. The observed quasi-reversible reductions of **3**–**7** in the range of −1.47 to −1.63 V are assigned to the reduction of the macrocyclic ring system (Fig. 7). Except for **1**, the first reduction potentials of **3**–**7** are cathodically shifted by 40–170 mV compared to axially alkoxy-disubstituted SiPcs (E_red_ = −990 mV)^42^.

The redox gap, ΔE_redox_, spans only 60 mV across the series of SiPcs and remains nearly constant at 1.69–1.75 V since axial substitution modulates these potentials in the same direction (Table 1). A similar behavior of the redox potentials and the near-constancy of the redox gap have been noted previously for other series of Pc complexes^26^.

### Photophysics

**Figure 9:**
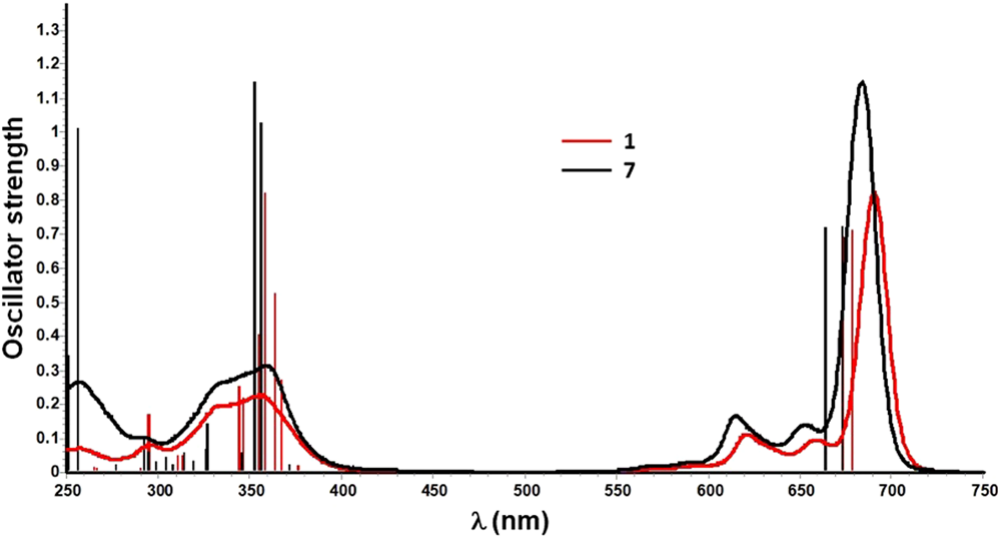
Typical overlays of experimental UV-vis absorption spectra (curved lines) of **1** and **7** with their predicted transitions (vertical bars) calculated by TD-DFT at room temperature in dichloromethane.

The UV-vis absorption spectra of all the SiPcs were recorded in dichloromethane and values are collated in Table 2. An overlay of the experimental UV-vis absorption spectra of **1** and **7** with their predicted transitions calculated by time-dependent DFT (TD-DFT) at room temperature in dichloromethane is shown in Fig. 9 and similar overlays of the experimental and TD-DFT predicted UV-vis profiles for SiPcs **1**–**7** are shown in Supplementary Figure S1. The absorption spectra exhibit profiles that are typical of the phthalocyanine chromophore; a B (or Soret) band at 355–360 nm, an intense and sharp near-infrared Q band at 682–691 nm, together with two vibronic bands at 613–621 nm and 651–659 nm. The TD-DFT-assigned characterisations of these bands are tabulated in Table 3. The Q-bands have molar absorption extinction coefficients, *ε*, of (26–29) × 10^4^ M^−1^ cm^−1^ (Table 3). For all the SiPcs the absorptivity of the Q band strictly follows the Beer-Lambert law as a function of concentration, thereby indicating that the compounds are essentially free from aggregation in dichloromethane^43^. Furthermore, the absence of either a red-shifting of the Q band at *ca*. 750 nm and a blue-shifting Q band at *ca*. 630 nm, excludes the formation of J and H aggregates, respectively. The retention of monomeric behaviour even at 10^–4^ M demonstrates that the axial ligands are very effective in sterically isolating the chromophoric Pc rings, by preventing intermolecular π stacking interactions between the macrocycles in solution^38^. The absorption spectra have a window between 420–560 nm, which is typical of phthalocyanines. The absorption cut-off (optical band gap) of the SiPcs is at 720–730 nm (1.72–1.70 eV). The TD-DFT calculations of **1**–**7** predict that the bands found between 257–350 nm are principally π → π* locally excited (LE) transitions within the conjugated macrocycle, whereas the π → π* LE bands of the aromatic carboxylates in **6** and **7** are superimposed with those of the phthalocyanine. The B bands at *ca*. 355 nm for **1** and **2** are due to the π → π* based transitions within the isoindole moieties of the macrocycle, whereas, these same bands in **3–7** also contain small contributions from n → π* transitions from the bridging N-atoms connecting the four isoindole moieties to the π* orbital of the isoindole (Table 3). For **6**, this band contains an additional π → π* based ligand-to-ligand charge transfer (LLCT) transition emanating from the 3,5-bis(trifluoromethyl)phenylcarboxylate to the macrocycle. The Q-band for all the SiPcs is predicted to originate from π → π* LE transitions among the isoindole moieties of the macrocycle. Fluorination of the carboxylate ligand from **L3** to **L4** leads to a modest red-shift of 3 nm in the Q band of **4** compared to that of **3**; similar behavior exists between **6** and **7**. The position of the Q band, which relates to the HOMO-LUMO gap, remains nearly constant at 682–691 nm across the series. Therefore, the HOMO-LUMO energy gap is invariant to the original of the axial substitution of the carboxyaltes, an observation corroborated by their electrochemistry. However, the lowest energy absorption maxima of these complexes are red-shifted by *ca*. 10–20 nm compared to those of alkoxy- and siloxy-substituted SiPcs^43–45^.

**Table 3:** Principal transitions by TD-DFT involved in the B and Q bands of **1**–**7**.

Cmpd	λ_abs_ /nm (ε /10^4^ M^−1^ cm^−1^)	λ_DFT_ /nm (f)	Major transition	Character
**1**	356 (7.8)	358 (0.8221)	H-6 → L (80%)	isoindole(π) to isoindole(π*)
	691 (29)	679 (0.7111)	H → L (98%)	isoindole(π) to isoindole(π*)
**2**	355 (7.6)	358 (0.8629)	H-5 → L (80%)	isoindole(π) to isoindole(π*)
	690 (27)	680 (0.701)	H → L (98%)	isoindole(π) to isoindole(π*)
**3**	358 (7.2)	353 (1.1026)	H-5 → L + 1 (90%)	isoindole(π) to isoindole(π*) (major) + bridged-N (l.p.) to isoindole(π*) (minor)
	682 (27)	672 (0.7208)	H → L (98%)	isoindole(π) to isoindole(π*)
**4**	358 (7.4)	357 (1.1138)	H-4 → L (83%)	isoindole(π) to isoindole(π*) (major) + bridged-N (l.p.) to isoindole(π*) (minor)
	685 (26)	674 (0.7187)	H → L (98%)	isoindole(π) to isoindole(π*)
**5**	360 (6.5)	354 (1.0757)	H-5 → L + 1 (89%)	isoindole(π) to isoindole(π*) (major) + bridged-N (l.p.) to isoindole(π*) (minor)
	683 (26)	672 (0.7072)	H → L (98%)	isoindole(π) to isoindole(π*)
**6**	358 (7.8)	358 (1.1374)	H-5 → L (71%)	isoindole(π) to isoindole(π*) (major) + bridged-N (l.p.) to isoindole(π*) (minor) + **L6**(π) to **L6**(π*)
	688 (27)	677 (0.7206)	H → L (98%)	isoindole(π) to isoindole(π*)
**7**	359 (7.2)	353 (1.1494)	H-9 → L + 1 (89%)	isoindole(π) to isoindole(π*) (major) + bridged-N (l.p.) to isoindole(π*) (minor)
	684 (27)	673 (0.7249)	H → L (98%)	isoindole(π) to isoindole(π*)
**R1**	360 (7.1)	356 (0.9739)	H-5 → L (85%)	isoindole(π) to isoindole(π*) (major) + bridged-N (l.p.) to isoindole(π*) (minor)
	683 (26)	672 (0.7350)	H → L (98%)	isoindole(π) to isoindole(π*)
**R2**	359 (7.1)	353 (1.1235)	H-9 → L + 1 (89%)	**L9**(π) to isoindole(π*) (major) + isoindole (π) to isoindole(π*) (minor)
	685 (27)	672 (0.7090)	H → L (98%)	isoindole(π) to isoindole(π*)

^a^TDDFT calculations were performed with the B3LYP/SBKJC-VDZ basis set for Si(IV) and 6–31 G** for C, H, N, and O, using a CPCM (dichloromethane) solvent model.

**Figure 10:**
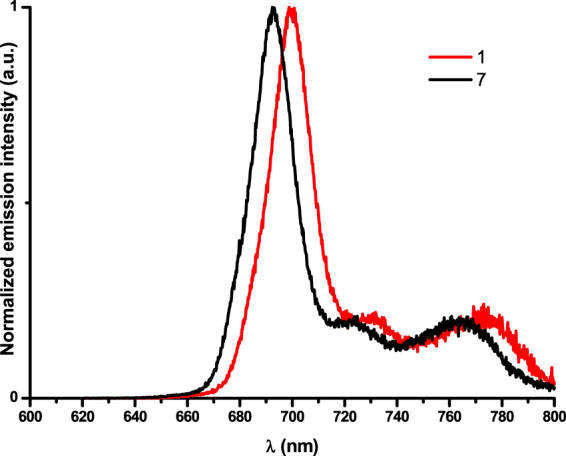
Typical normalized emission spectra of complexes **1** and **7** in degassed dichloromethane solutions at room temperature (λ_exc_ = 360 nm).

The SiPcs **1**–**7** were also characterized by photoluminescence spectroscopy in dilute and degassed dichloromethane solutions at ambient temperature. Relevant photophysical data are shown in Table 4 and the photoluminescence spectra of SiPcs **1** and **7** are shown in Fig. 10, whereas the photoluminescence spectra of all the SiPcs in dichloromethane solutions are shown in Supplementary Figure S2. All the SiPcs emit strongly in the near-infrared (NIR) region with *λ*_em_ ranging narrowly from 688–700 nm. Two lower intensity bands are observed at between 710–790 nm. The photoluminescence spectrum of each of the SiPcs is a mirror image of the absorption spectrum. The *λ*_em_ values are typical of carboxy-substituted SiPc compounds and the emission is modestly red-shifted by *ca*. 8–30 nm compared to alkoxy- and silyloxy-disubstituted SiPcs. All the SiPcs exhibit short mono-exponential photoluminescence decay kinetics (τ) in the range 6.7–7.3 ns and high photoluminescence quantum yields (Φ_PL_ = 40–52%) in dichloromethane solution. The calculated radiative lifetime, τ_rad_ = τ × Φ_PL_^−1^, ranges from 13.8–18 ns and the radiative, *k*_r_, and non-radiative, *k*_nr_, decay constants, [*k*_r_ = Φ_PL_/τ_ε_ and *k*_nr_ = (*k*_r_/Φ_PL_) − *k*_r_] are in the range of (55.5–72.2) × 10^6^ and (66.7–83.3) × 10^6^ s^−1^ for all the compounds. The mirror image behavior between absorption and emission spectra, small Stokes shift (*λ*_abs_ − *λ*_em_, of 6–10 nm), short excited-state decay and radiative lifetimes are all consistent with a spin-allowed fluorescence process from the S_1_ state. Unlike the strong emission quenching observed for amino-alkoxy-substituted SiPcs, produced by intramolecular photoinduced electron transfer (PET)^44,46^, the presence of the carboxylate ligands is benign to the radiative decay of the excited state. In general, for the SiPcs with electron-donating groups, higher fluorescence intensity was observed compared to those with electron-withdrawing groups, Table 4)^47^. The higher Φ_PL_ values of **3** *vs*. **4** and **6** *vs*. **7** are the result of a decrease in *k*_nr_ of the respective non-fluorinated SiPcs, **3** and **6** *vs*. the fluorinated SiPcs, **4** and **7**.

**Table 4:** Photophysical data of complexes **1–7** ^a^.

SiPc	*λ*_em_/nm	Φ_PL_/%^b^	*τ*/ns	*τ*_rad_/ns^c^	*k*_r_/× 10^6^s^−1d^	*k*_*n*r_/× 10^6^ s^−1e^
1	700	42	7.3	17.4	57.5	79.4
2	697	48	6.7	13.9	71.6	77.6
3	688	52	7.2	13.8	72.2	66.7
4	692	46	6.9	15.0	66.7	78.3
5	692	41	7.3	17.8	56.2	80.8
6	698	40	7.2	18.0	55.5	83.3
7	693	43	7.2	16.7	59.7	79.2

^a^In dichloromethane solution at room temperature. ^b^Determined using relative method using **R2** as the reference. ^c^Calculated from *τ*_rad_ = τ × Φ^−1^. ^d^Calculated from *k*_r_ = Φ_PL_/τ_ε_. ^e^Calculated from *k*_nr_ = (*k*_r_/Φ_PL_) − *k*_r_.

Thin-film photoluminescence studies of the SiPcs **1**–**7** were performed in a PMMA host and the corresponding PL emission maxima, photoluminescence quantum yields and excited-state lifetime values are compiled in Table 5. The concentration of the SiPcs in the PMMA matrix were maintained at an optical density below 0.1 to avoid any concentration quenching effects. The PL emission maxima of SiPcs **1**–**7** in doped PMMA overlap nicely with their respective solution PL maxima, suggesting that in both thin film and solution states aggregation induced red-shifting of the PL maximum is not evident. The film PL quantum yield values of the SiPcs remain very similar to those observed in solution, except for SiPc **6** which shows higher PLQY in the film. The higher PLQY of SiPc **6** seems to be associated with a higher rate of radiative decay constant (*k*_*r*_) of 81.8 × 10^6^ s^−1^ compared to the *k*_r_ values of the other SiPcs (66.1–80.0 × 10^6^ s^−1^).

**Table 5:** Photophysical data of complexes **1–7** in PMMA doped film^a^.

SiPc	*λ*_em_/nm	Φ_PL_/%^b^	*τ*/ns	*τ*_rad_/ns^c^	*k*_r_ / × 10^6^s^−1d^	*k*_*n*r_/ × 10^6^ s^−1e^
**1**	700	46	6.3	13.7	73.0	85.7
**2**	697	43	6.3	14.6	68.2	90.4
**3**	687	56	7.0	12.5	80.0	62.8
**4**	692	45	6.3	14.0	71.4	87.3
**5**	691	46	6.2	13.5	74.2	87.1
**6**	698	54	6.6	12.2	81.8	69.7
**7**	693	41	6.2	15.1	66.1	95.1

^a^In PMMA at room temperature (λ_exc_ = 612 nm). ^b^Determined using an integrating sphere. ^c^Calculated from *τ*_rad_ = τ × Φ^−1^. ^d^Calculated from *k*_r_ = Φ_PL_/τ. ^e^Calculated from *k*_nr_ = (*k*_r_/Φ_PL_) −*k*_r_.

## Conclusions

In summary, a series of SiPcs bearing axially substituted carboxylate ligands have been synthesized and structurally and optoelectronically characterized. Their structures were unambiguously characterized by ^1^H NMR spectroscopy, ESI-MS, elemental analyses and single crystal XRD analyses. The presence of electron-withdrawing or donating groups on the carboxylate ligands does not exert any significant influence on the optoelectronic properties of the SiPcs as the electron-donating/withdrawing groups exert similar effects upon the phthalocyanine ring based HOMO and LUMO. The oxidation and the reduction processes of the complexes were both found to be quasi-reversible. All the SiPcs exhibit NIR fluorescence with high photoluminescence quantum yields and no sign of aggregation in dilute solution. This study demonstrates the wide selection of carboxylate ligands available to render the SiPc compounds solution-processable and thus amenable for use in OLED and OPV devices.

## Experimental Section

### General Synthetic Procedure

Commercial chemicals were used as supplied. Chromatography was performed on columns with an i.d. of 25–30 mm on silica gel. The reaction was performed under nitrogen using standard Schlenk techniques. Purification and handling of the products were carried out under air. The progress of reactions and the elution of products were followed on TLC plates (silica gel on aluminum sheets, 250 µm with indicator F-254). Compounds were visualized under UV light. ^1^H and ^19^F NMR spectra were recorded with Bruker AVANCE 300 (300 MHz for ^1^H; 282 MHz for ^19^F) and Bruker AVANCE II 400 spectrometers (400 MHz for ^1^H; 377 MHz for ^19^F). The following abbreviations have been used for multiplicity assignments: “s” for singlet, “d” for doublet, “t” for triplet, “m” for multiplet, and “br” for broad. ^1^H NMR spectra were referenced to the solvent peak. Melting points (m.p.) were recorded in open-end capillaries on an Electrothermal melting point apparatus IA9200 and are uncorrected. The heating rate was 0.5 or 1.0 °C/min. High-resolution nanospray ionization (HR NSI^+^) and MALDI^+^ mass spectra, were recorded at the EPSRC National Mass Spectrometry Service Centre, Swansea University. Elemental analyses were performed by Mr. Stephen Boyer, London Metropolitan University.

Silicon phthalocyanine dichloride (SiPcCl_2_; dye content ~85%) and carboxylic acid (excess) were stirred in 3–4 mL of dry and degassed DMF or diglyme (degassed by bubbling with nitrogen for 5 min) at 140–165 °C overnight to give dark green-blue solution or suspension. The volume of the solvent was chosen to be as small as possible, while still allowing the efficient mixing of the reagents. The temperature of the reaction was as high as possible to help dissolve the starting materials; however, the temperature was lowered for the fluorinated acids to prevent side-reactions involving the electron-deficient C–F bonds. The molar ratio of SiPcCl_2_ to carboxylic acid ranged from 3.9–7.4. The reaction mixture was cooled to room temperature. Water (50 mL or more) was added to precipitate the product (blue solid). For **2**, addition of water precipitated a dark oil; the supernatant solution was decanted and the oil was crystallized on mixing with methanol/water (1/1, v/v). The solid product was filtered. The product was washed on the filter with water for **6**; with water and hexane (5–10 mL) for **3**; or with water and methanol/water (1/1, v/v) for **1**, **2**, **4**, **5** and **7**. The washing was performed to remove the reaction solvents and the excess of the acid. The product was purified by column chromatography on 10–22 g of silica. The elution was performed with dichloromethane (except for **4**) or with 0–0.3% methanol in dichloromethane (for **4**) to give green-blue eluate of the product as the first band. The combined product fractions were evaporated to dryness. The product was dried under vacuum.

The products are air- and moisture stable blue solids that are soluble in chlorinated solvents (chloroform, dichloromethane, 1,2-dichlorobenzene) and are insoluble in acetonitrile. The exact shade, brightness, and depth of the blue color of the product mainly depend on whether the product is crystalline or not. The crystals of the product may look red in reflection. The products were stored in the dark. Further details are provided below.

1. SiPcCl_2_ (100 mg, 0.16 mmol, 1 equiv.) and perfluoroheptanoic acid (286 mg, 0.79 mmol, 4.94 equiv.) in diglyme at 140 °C gave 87 mg (0.069 mmol, 43%) of **1**. M.p.: 314 °C (dec.). ^1^H NMR (300 MHz, CDCl_3_): *δ* = 9.72 (m, 8 H), 8.45 (m, 8 H) ppm. ^19^F NMR (377 MHz, CDCl_3_): *δ* = −81.01 (m, 6 F), −121.22 (t, *J* = 11 Hz, 4 F), −123.77 (m, 8 H), −125.55 (m, 4 H), −126.80 (m, 4 H) ppm. HR NSI^+^ MS: *m/z* 1267.0721 ({M + H}^+^, 100%); theor. C_46_H_17_F_26_N_8_O_4_Si^+^ *m/z* 1267.0721. Anal. Calcd for C_46_H_16_F_26_N_8_O_4_Si (MW 1266.73): C, 43.62; H, 1.27; N, 8.85. Found: C, 43.64; H, 1.22; N, 8.76.
2. SiPcCl_2_ (100 mg, 0.16 mmol, 1 equiv.) and perfluoro-3,7-dimethyloctanoic acid (351 mg, 0.68 mmol, 4.25 equiv.) in diglyme at 140 °C gave 7 mg (0.004 mmol, 3%) of **2**. ^1^H NMR (300 MHz, CDCl_3_): *δ* = 9.71 (m, 8 H), 8.44 (m, 8 H) ppm. ^19^F NMR (282 MHz, CDCl_3_): *δ* = −72.36 (m, CF_3_), −113.75 (m, CF_2_), −115.55 (m, CF_2_), −121.05 (m, CF_2_), −186.72 (m, CF), −187.20 (m, CF) ppm. HR NSI^+^ MS: *m/z* 1567.0519 ({M + H}^+^, 100%); theor. C_52_H_17_F_38_N_8_O_4_Si^+^ *m/z* 1567.0530.
3. SiPcCl_2_ (200 mg, 0.33 mmol, 1 equiv.) and pivalic acid (250 mg, 2.45 mmol, 7.42 equiv.) in *N,N′*-DMF gave 151 mg (0.20 mmol, 59%) of **3**. M.p.: >380 °C. ^1^H NMR (400 MHz, CDCl_3_): *δ* = 9.69 (m, 8 H), 8.38 (m, 8 H), −1.30 (s, 18 H) ppm. ^1^H NMR (400 MHz, CD_2_Cl_2_): *δ* = 9.72 (m, 8 H), 8.43 (m, 8 H), −1.32 (s, 18 H) ppm. HR NSI^+^ MS: *m/z* 765.2363 ({M + Na}^+^, 6%), 743.2546 ({M + H}^+^, 1%); theor. C_42_H_34_N_8_O_4_SiNa^+^ *m/z* 765.2364; theor. C_42_H_35_N_8_O_4_Si^+^ *m/z* 743.2545. Anal. Calcd for C_42_H_34_N_8_O_4_Si·1.5H_2_O (MW 769.90): C, 65.52; H, 4.84; N, 14.55. Found: C, 65.69; H, 4.57; N, 14.24.
4. SiPcCl_2_ (100 mg, 0.16 mmol, 1 equiv.) and 3,3,3-trifluoro-2,2-dimethylpropionic acid (163 mg, 1.04 mmol, 6.5 equiv.) in diglyme at 160 °C gave 87 mg (0.10 mmol, 64%) of **4**. M.p.: >380 °C. ^1^H NMR (300 MHz, CD_2_Cl_2_): *δ* = 9.74 (m, 8 H), 8.46 (m, 8 H), −0.98 (s, 12 H) ppm. ^19^F NMR (282 MHz, CD_2_Cl_2_): *δ* = −77.9 ppm. HR NSI^+^ MS: *m/z* 851.1982 ({M + H}^+^, 100%); theor. C_42_H_29_F_6_N_8_O_4_Si^+^ *m/z* 851.1980. Anal. Calcd for C_42_H_28_F_6_N_8_O_4_Si (MW 850.81): C, 59.29; H, 3.32; N, 13.17. Found: C, 59.61; H, 3.20; N, 12.97.
5. SiPcCl_2_ (100 mg, 0.16 mmol, 1 equiv.) and 1-adamantanecarboxylic acid (133 mg, 0.98 mmol, 6.12 equiv.) in diglyme at 160 °C gave 52 mg (0.058 mmol, 36%) of **5**. M.p.: >380 °C. ^1^H NMR (300 MHz, CDCl_3_): *δ* = 9.69 (m, 8 H), 8.37 (m, 8 H), 0.76 (m, 12 H), 0.39 (m, 6 H), −0.95 (s, br, 12 H) ppm. HR NSI^+^ MS: *m/z* 921.3306 ({M + Na}^+^, 12%); theor. C_54_H_46_N_8_O_4_SiNa^+^ *m/z* 921.3303. Anal. Calcd for C_54_H_46_N_8_O_4_Si (MW 899.10): C, 72.14; H, 5.16; N, 12.46. Found: C, 72.11; H, 4.95; N, 12.37.
6. SiPcCl_2_ (100 mg, 0.16 mmol, 1 equiv.) and 3,5-bis(trifluoromethyl)benzoic acid (169 mg, 0.65 mmol, 4.06 equiv.) in diglyme at 160 °C gave 72 mg (0.068 mmol, 43%) of **6**. M.p.: >350 °C (dec.).^1^H NMR (300 MHz, CDCl_3_): *δ* = 9.74 (m, 8 H), 8.43 (m, 8 H), 7.09 (s, br, 2 H), 5.47 (d, *J* = 1.0 Hz, 4 H) ppm. ^19^F NMR (282 MHz, CDCl_3_): *δ* = −64.4 ppm. ^1^H NMR (300 MHz, CD_2_Cl_2_): *δ* = 9.75 (m, 8 H), 8.47 (m, 8 H), 7.12 (s, 2 H), 5.48 (s, 2 H) ppm. ^19^F NMR (282 MHz, CD_2_Cl_2_): *δ* = −64.8 ppm. HR NSI^+^ MS: *m/z* 1077.1239 ({M + Na}^+^, 30%); theor. C_50_H_22_F_12_N_8_O_4_SiNa^+^ *m/z* 1077.1234. Anal. Calcd for C_50_H_22_F_12_N_8_O_4_Si (MW 1054.84): C, 56.93; H, 2.10; N, 10.62. Found: C, 56.55; H, 2.08; N, 10.51.
7. SiPcCl_2_ (100 mg, 0.16 mmol, 1 equiv.) and 4-octyloxybenzoic acid (164 mg, 0.66 mmol, 4.12 equiv.) in diglyme at 160 °C gave 41 mg (0.039 mmol, 25%) of **7**. M.p.: 307–309 °C. ^1^H NMR (400 MHz, CDCl_3_): *δ* = 9.70 (m, 8 H), 8.37 (m, 8 H), 5.69 (d, *J* = 9.2 Hz, 4 H), 5.04 (d, *J* = 9.0 Hz, 4 H), 3.34 (t, *J* = 6.6 Hz, 4 H), 1.33 (m, 4 H), 1.14 (m, 4 H), 1.06 (m, 16 H), 0.76 (t, *J* = 6.9 Hz, 6 H) ppm. MALDI^+^ MS: *m/z* 1038.4 (M^+^, 90%); theor. C_62_H_58_N_8_O_6_Si^+^ *m/z* 1038.4. Anal. Calcd for C_62_H_58_N_8_O_6_Si (MW 1039.28): C, 71.65; H, 5.63; N, 10.78. Found: C, 71.77; H, 5.53; N, 10.78.

### Photophysical measurements

The solutions for photophysical investigation were prepared in HPLC-grade dichloromethane. Electronic absorption spectra were recorded with a Shimadzu UV-1800, UV-VIS Spectrophotometer in optical quartz cells of 10 mm path length. The absorption spectra were recorded on a PerkinElmer LAMBDA 950 UV/Vis/NIR spectrophotometer. The photoluminescence spectra were recorded on an Edinburgh Instruments FLS-980 fluorescence spectrometer and were corrected for the spectral sensitivity of the detection system. In dilute solutions used in the photophysical measurements, the photoluminescence spectra were independent of the excitation wavelength. In our earlier work, the absolute photoluminescence quantum yields (PLQY) of SiPc carboxylates **R1** and **R2** were determined using an integrating sphere on a Hamamatsu C9920–02 absolute PLQY measurement system^32^. Photoluminescene quantum yields for the SiPcs in this work were determined by a relative method using **R2** as the standard. The photoluminescence lifetime was measured using the time-correlated single-photon-counting technique integrated in the FLS-980 fluorescence spectrometer. The samples were excited at 375 nm by pico-second pulsed diode laser head Picoquant LDH-D-C-375. The luminescence decay was measured at a wavelength corresponding to the emission peak. The emitted light was dispersed in a monochromator with an entrance slit of 2–4 nm and was detected on a microchannel plate detector (<100 ps response). The luminescence decay was fitted to a mono-exponential function with aχ^2^ value in the range of 1–1.25.

### Electrochemistry

Electrochemical experiments were conducted in HPLC grade dichloromethane with 0.1 M tetra-*n*-butylammonium hexafluorophosphate as the supporting electrolyte, with a PC controlled electrochemical workstation (Electrochemical Analyzer/Workstation CHI600C, CH Instruments, Inc.) at room temperature.

The experiments were carried out under nitrogen in an electrochemical cell through which a stream of nitrogen was passed. The volume of the solvent was 2–3 mL. Glassy-carbon disk (3 mm diameter), platinum spiral, and platinum wire served as working, counter, and quasi-reference electrodes, respectively. Estimated error: ±30 mV. Cyclic voltammetry was performed at scan rates of 0.1 V/s and 1 V/s. The potentials of reversible redox processes were independent of the scan rate.

### DFT calculations

All calculations were performed with the Gaussian09, revision D.01^48^ suite of programs employing the DFT method, the Becke three-parameter hybrid functional^49^, and Lee-Yang-Parr’s gradient-corrected correlation functional (B3LYP)^50^. Singlet ground state geometry optimizations and single point energy calculations for **1**–**7**, **R1** and **R2** were carried out at the (R)B3LYP levels, using their respective crystallographic structures as starting points. All elements except Silicon were assigned the 6–31 G(d,p) basis set^51^. The double-ζ quality SBKJC VDZ ECP basis set^52^ with an effective core potential was employed for the Si(VI)-ion. Vertical electronic excitations based on (R)B3LYP-optimized geometries were computed for **1**–**7**, **R1** and **R2** using the TD-DFT formalism^53,54^ in dichloromethane using conductor-like polarizable continuum model (CPCM)^55–57^. Vibrational frequency calculations were performed to ensure that the optimized geometries represent the local minima and there are only positive eigenvalues. The electronic distribution and localization of the singlet excited states were visualized using the electron density difference maps (ED-DMs)^58^. *Gausssum 2.2* and *Chemissian* v3.8^59^ were employed to visualize the absorption spectra (simulated with Gaussian distribution with a full-width at half maximum (fwhm) set to 3000 cm^−1^) and to calculate the fractional contributions of various groups to each molecular orbital. All calculated structures and Kohn-Sham orbitals were visualized with ChemCraft^60^.

### X-ray Crystallography

Diffraction data for compounds **1** and **3**–**7** were collected by using a Rigaku FR-X Ultrahigh brilliance Microfocus RA generator/confocal optics and Rigaku XtaLAB P200 system, using Mo Kα radiation (λ = 0.71073 Å). Data for compound **2** were collected by using a Rigaku MM-007HF High brilliance RA generator/confocal optics and Rigaku XtaLAB P100 system, using Cu Kα radiation (λ = 1.54187 Å). Intensity data for all structures were collected at 173 K, using either ω, or both ω and φ steps accumulating area detector images spanning at least a hemisphere of reciprocal space. All data were corrected for Lorentz polarization effects. A multiscan absorption correction was applied by using CrystalClear^61^. Structures were solved by direct (SIR2004^62^ or SIR2011^63^) methods and refined by full-matrix least-squares against F^2^ (SHELXL-2013)^64^. Non-hydrogen atoms were refined anisotropically, and hydrogen atoms were refined using a riding model. All calculations were performed using the CrystalStructure^65^ interface. Data for **2** showed positional disorder throughout the perfluoroalkyl chain that could not be modelled. This resulted in the unusually large thermal ellipsoids seen for these atoms, and necessitated the use of a range of restraints to bond distances and thermal ellipsoids. Crystallographic data are presented in Table 1.

### Data availability

The data supporting this study are available at: http://dx.doi.org/10.17630/ea33220b-53f6-4503-84d0-ae052914ecff.

## Electronic supplementary material

ESI
