# Supplementary material for: Near-Infrared Fluorescence of Silicon Phthalocyanine Carboxylate Esters
Source: Sci Rep. 2017 Sep 25;7:12282. doi: 10.1038/s41598-017-12374-8 (PMC5612943; doi:10.1038/s41598-017-12374-8)
Supplement: Supplementary file 1 — ESI [file 41598_2017_12374_MOESM1_ESM.pdf]

Near-Infrared Fluorescence of Silicon Phthalocyanine Carboxylate Esters

Amlan K. Pal,[a] Shinto Varghese,[b] David B. Cordes,[c] Alexandra M. Z. Slawin,[c] Ifor D. W. Samuel[b] and Eli Zysman-Colman*,[a]

[a] Organic Semiconductor Centre, EaStCHEM School of Chemistry, University of St Andrews, St Andrews, Fife, UK, KY16 9ST, Fax: +44-1334 463808; Tel: +44-1334 463826; E-mail: eli.zysman-colman@st-andrews.ac.uk;
URL: http://www.zysman-colman.com
[b] Organic Semiconductor Centre, SUPA School of Physics and Astronomy, University of St Andrews, St Andrews, Fife, UK, KY16 9SS
[c] EaStCHEM School of Chemistry, University of St. Andrews, St. Andrews, KY16 9ST, Fife, United Kingdom

SUPPLEMENTARY INFORMATION

Table of Contents: **Pages**

Supplementary Table S1 S2
Supplementary Table S2 S3
Supplementary Figure S1 S3
Supplementary Figure S2 S4
Supplementary Tables S3-S11 S4-S26
Reference S26

Supplementary Table S1. Crystal Data and Structure Refinement.

compound	**1**	**2**	**3**	**4**	**5**	**6**	**7**
empirical formula	$C_{46}H_{16}F_{26}N_8O_4Si$	$C_{52}H_{16}F_{38}N_8O_4Si$	$C_{42}H_{38}N_8O_4Si$	$C_{42}H_{28}F_6N_8O_4Si$	$C_{54}H_{46}N_8O_4Si$	$C_{52}H_{25}F_{12}N_9O_4Si$	$C_{62}H_{58}N_8O_6Si$
formula weight	1266.73	1566.77	742.87	850.81	899.09	1095.89	1039.28
crystal appearance	blue platelet	blue platelet	blue platelet	blue platelet	blue platelet	blue platelet	blue platelet
crystal size [mm^3]	0.24×0.10×0.01	0.24×0.04×0.01	0.15×0.12×0.02	0.10×0.06×0.02	0.18×0.07×0.02	0.24×0.06×0.01	0.36×0.12×0.01
space group	$P\bar{1}$	$P\bar{1}$	$P2_1/c$	$P2_1/c$	$P\bar{1}$	$P2_1/c$	$P2_1/c$
a [Å]	8.932(2)	9.3320(16)	10.4400(12)	10.7748(11)	9.3865(8)	22.380(4)	15.229(8)
b [Å]	11.534(3)	10.952(2)	19.233(2)	18.7760(19)	11.3937(8)	47.574(9)	20.881(8)
c [Å]	12.663(3)	15.174(4)	9.7263(11)	9.9159(10)	12.0503(11)	8.8602(18)	8.404(4)
α [°]	90.138(3)	82.208(13)			117.465(5)		
β [°]	105.564(5)	81.253(11)	110.931(3)	112.471(3)	106.875(11)	95.808(2)	100.133(11)
γ [°]	111.498(5)	66.080(12)			92.068(9)		
volume [Å]^3	1161.9(5)	1396.5(5)	1824.1(4)	1853.7(3)	1072.11(19)	9385(3)	2631(2)
Z	1	1	2	2	1	8	2
ρ (calc) [g/cm^3]	1.810	1.863	1.352	1.524	1.392	1.551	1.312
μ [mm^{-1}]	0.213	2.091	0.121	0.152	0.116	0.157	0.107
reflections collected	14243	13837	22142	22471	13288	85393	49736
Independent reflections (R_{int})	4213 (0.0438)	4956 (0.0907)	3344 (0.0438)	3393 (0.04196)	3895 (0.0286)	16192 (0.0381)	4833 (0.3474)
GoF on F^2	1.012	1.942	1.012	1.060	1.074	1.040	1.085
R_1, wR_2 [$I > 2\sigma(I)$]	0.0435, 0.1043	0.2339, 0.5627	0.0333, 0.0892	0.0346, 0.0926	0.0336, 0.0909	0.0469, 0.1257	0.1602, 0.3645
[illegible]	[illegible]	[illegible]	[illegible]	[illegible]	[illegible]	[illegible]	[illegible]

Supplementary Table S2. Selected bond distances of SiPcs **1-7**, **R1** and **R2**.

SiPc	Bond lengths (Å)			Shortest Si···Si
	Si–O	Si–N	Si–N	
**1**	1.7794(16)	1.8897(17)	1.900(2)	8.932(2)
**2**	1.757(10)	1.891(9)	1.899(11)	9.3320(16)
**3**	1.7459(10)	1.9022(13)	1.9095(10)	9.7263(11)
**4**	1.7549(11)	1.9029(14)	1.9031(10)	9.9159(10)
**5**	1.7358(13)	1.9044(11)	1.9203(12)	9.3865(8)
**6** (Si1)	1.7554(17)	1.8989(19)	1.9003(19)	8.8602(18)
	1.7581(16)	1.900(2)	1.902(2)	
(Si51)	1.7583(16)	1.900(2)	1.9034(19)	
	1.7623(16)	1.9020(19)	1.905(2)	
**7**	1.736(7)	1.898(10)	1.915(7)	8.404(4)
**R1**[a]	1.7472(9)	1.9065(11)	1.9161(16)	9.1436(15)
**R2**[a] (Si1)	1.7485(17)	1.906(2)	1.913(2)	11.313(2)
(Si41)	1.7518(17)	1.898(2)	1.9188(19)	

[a]Taken from Ref. [1]

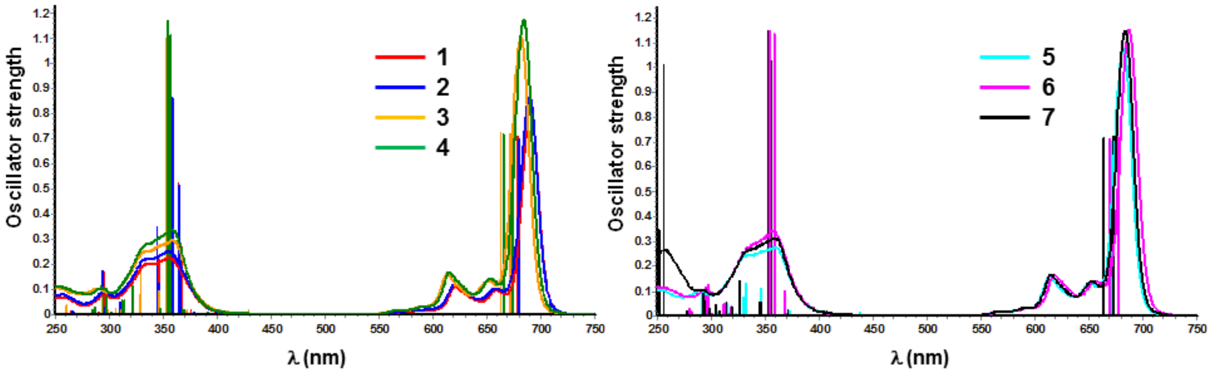

Supplementary Figure S1: Overlays of experimental UV-vis absorption spectra (curved lines) of complexes **1-7** with their predicted transitions (vertical bars) calculated by singlet TD-DFT at room temperature in dichloromethane.

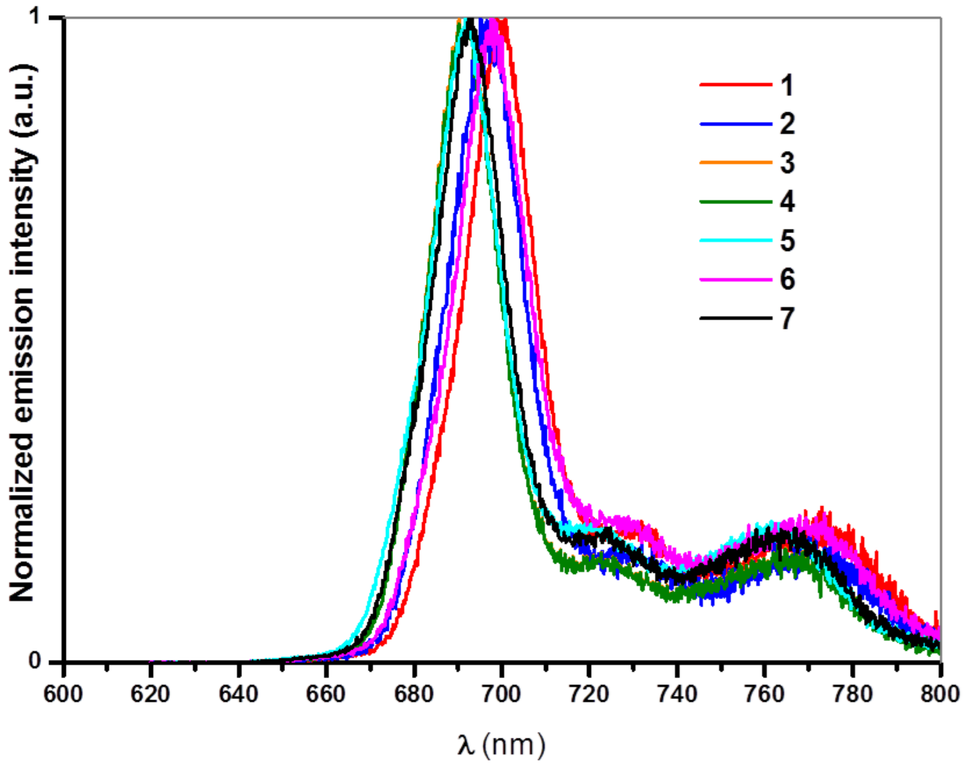

Supplementary Figure S2: Normalized emission spectra of complexes **1-7** in degassed dichloromethane solutions at room temperature (λ_{exc} = 360 nm).

Supplementary Table S3: DFT optimised atomic coordinates of SiPc **1**.

Center Number	Atomic Number	Atomic Type	Coordinates (Angstroms) X	Y	Z
1	14	0	-0.000055	-0.000011	-0.000054
2	9	0	-3.064223	-1.010547	4.121946
3	9	0	-1.936809	0.878862	3.978899
4	9	0	-4.567717	1.149076	3.648944
5	9	0	-3.415533	1.645973	1.843874
6	9	0	-5.423854	-1.124092	2.649272
7	9	0	-3.962404	-1.065460	0.997563
8	9	0	-5.115714	1.040948	-0.100725
9	9	0	-6.424827	1.359844	1.643022
10	9	0	-7.787820	-0.788581	1.208364
11	9	0	-6.271008	-1.671081	-0.123900
12	9	0	-8.630038	-0.661918	-1.259770
13	9	0	-6.744476	0.111327	-2.019419
14	9	0	-7.976993	1.340083	-0.701611
15	8	0	-1.046400	0.161881	1.499603
16	8	0	-1.421787	-1.940990	2.294238
17	7	0	-0.019060	-3.371285	-0.085388

18	7	0	-1.159189	-1.342029	-0.771349
19	7	0	-2.816047	0.064379	-1.850361
20	7	0	-1.112527	1.395660	-0.752679
21	6	0	-0.989285	-2.714107	-0.686197
22	6	0	-2.307360	-1.101557	-1.505397
23	6	0	-2.913251	-2.363034	-1.877527
24	6	0	-2.077832	-3.374460	-1.378968
25	6	0	-4.090724	-2.662774	-2.566889
26	1	0	-4.745850	-1.872161	-2.916458
27	6	0	-4.393330	-4.008362	-2.757316
28	1	0	-5.300500	-4.284526	-3.286418
29	6	0	-3.546610	-5.025797	-2.269995
30	1	0	-3.817078	-6.063870	-2.438622
31	6	0	-2.381208	-4.724446	-1.570079
32	1	0	-1.731323	-5.499906	-1.179480
33	6	0	-2.263491	1.207871	-1.494598
34	6	0	-0.935130	2.757709	-0.583773
35	6	0	-2.000435	3.468054	-1.262888
36	6	0	-2.835664	2.495411	-1.832611
37	6	0	-2.275104	4.830357	-1.400300
38	1	0	-1.622665	5.575356	-0.958133
39	6	0	-3.412180	5.184446	-2.122011
40	1	0	-3.659000	6.233874	-2.252350
41	6	0	-4.257483	4.205801	-2.684713
42	1	0	-5.139736	4.521782	-3.233325
43	6	0	-3.983506	2.847632	-2.546291
44	1	0	-4.636673	2.086008	-2.958121
45	6	0	-1.598424	-0.743309	2.270809
46	6	0	-2.592296	-0.076600	3.264905
47	6	0	-3.831205	0.630169	2.638606
48	6	0	-4.750648	-0.309635	1.808393
49	6	0	-5.780831	0.427185	0.906626
50	6	0	-6.850213	-0.515179	0.277296
51	6	0	-7.566328	0.091923	-0.959644
52	9	0	3.064255	1.010338	-4.121978
53	9	0	1.936913	-0.879088	-3.978755
54	9	0	4.567847	-1.149181	-3.648835
55	9	0	3.415695	-1.646042	-1.843728
56	9	0	5.423908	1.124047	-2.649251
57	9	0	3.962399	1.065429	-0.997579
58	9	0	5.115872	-1.040815	0.100896
59	9	0	6.424951	-1.359794	-1.642853
60	9	0	7.787744	0.788916	-1.208468
61	9	0	6.271027	1.671134	0.124094
62	9	0	8.630257	0.662234	1.259615
63	9	0	6.744950	-0.111553	2.019345
64	9	0	7.977593	-1.339820	0.701204
65	8	0	1.046166	-0.161942	-1.499783
66	8	0	1.422176	1.940948	-2.294049
67	7	0	0.018941	3.371277	0.085280

68	7	0	1.159040	1.342010	0.771282
69	7	0	2.815841	-0.064391	1.850398
70	7	0	1.112407	-1.395662	0.752579
71	6	0	0.989152	2.714095	0.686101
72	6	0	2.307172	1.101544	1.505402
73	6	0	2.913031	2.363026	1.877571
74	6	0	2.077657	3.374446	1.378933
75	6	0	4.090466	2.662767	2.567000
76	1	0	4.745541	1.872156	2.916660
77	6	0	4.393087	4.008357	2.757392
78	1	0	5.300228	4.284527	3.286541
79	6	0	3.546420	5.025788	2.269974
80	1	0	3.816903	6.063863	2.438570
81	6	0	2.381049	4.724434	1.570007
82	1	0	1.731208	5.499893	1.179336
83	6	0	2.263320	-1.207882	1.494575
84	6	0	0.935011	-2.757716	0.583661
85	6	0	2.000291	-3.468063	1.262814
86	6	0	2.835474	-2.495423	1.832607
87	6	0	2.274961	-4.830365	1.400228
88	1	0	1.622561	-5.575362	0.958000
89	6	0	3.411988	-5.184453	2.122018
90	1	0	3.658811	-6.233881	2.252357
91	6	0	4.257236	-4.205810	2.684806
92	1	0	5.139441	-4.521792	3.233494
93	6	0	3.983257	-2.847643	2.546378
94	1	0	4.636370	-2.086012	2.958285
95	6	0	1.598533	0.743223	-2.270770
96	6	0	2.592384	0.076442	-3.264847
97	6	0	3.831324	-0.630254	-2.638519
98	6	0	4.750715	0.309597	-1.808340
99	6	0	5.780927	-0.427113	-0.906530
100	6	0	6.850313	0.515351	-0.277291
101	6	0	7.566692	-0.091786	0.959478

Supplementary Table S4: DFT optimised atomic coordinates of SiPc **2**.

Center Number	Atomic Number	Atomic Type	Coordinates (Angstroms) X	Y	Z
1	14	0	-0.000007	-0.000016	-0.000004
2	9	0	3.566117	2.171639	-3.135455
3	9	0	3.816397	0.130221	-2.346981
4	9	0	3.845438	1.325966	0.294705
5	9	0	6.412750	2.830189	-0.583384
6	9	0	6.028144	1.686139	-2.407618
7	9	0	6.389512	0.796964	0.961389

8	9	0	5.538552	-0.570334	-0.536374
9	9	0	7.559471	-0.484714	-2.085342
10	9	0	8.496687	1.231936	-1.096925
11	9	0	7.665596	-1.566881	0.821882
12	9	0	8.891741	1.001436	1.563097
13	9	0	9.830463	-0.885389	2.053701
14	9	0	10.600925	0.318121	0.406909
15	9	0	4.341812	4.240231	-1.607483
16	9	0	2.510340	3.616471	-0.604897
17	9	0	4.328631	3.869621	0.541194
18	9	0	8.539398	-2.775590	-1.321899
19	9	0	10.091236	-1.290194	-1.713128
20	9	0	10.203534	-2.564240	0.046784
21	8	0	1.559918	0.416436	-0.858621
22	8	0	1.044323	1.885916	-2.520322
23	7	0	-0.445181	-2.653852	-2.034168
24	7	0	-0.966480	-0.316102	-1.648447
25	7	0	-2.026037	1.843353	-1.963095
26	7	0	-0.617714	1.834078	0.012755
27	6	0	-1.036494	-1.514041	-2.336302
28	6	0	-1.777863	0.595219	-2.301160
29	6	0	-2.369645	-0.035686	-3.463874
30	6	0	-1.901944	-1.358353	-3.488317
31	6	0	-3.241824	0.444558	-4.442953
32	1	0	-3.597097	1.468856	-4.414831
33	6	0	-3.629699	-0.441060	-5.445114
34	1	0	-4.306826	-0.105354	-6.224807
35	6	0	-3.160325	-1.770624	-5.469354
36	1	0	-3.485559	-2.431664	-6.267102
37	6	0	-2.291253	-2.248457	-4.491988
38	1	0	-1.926108	-3.269744	-4.501288
39	6	0	-1.477346	2.408560	-0.908000
40	6	0	-0.316296	2.790695	0.965794
41	6	0	-0.993388	4.028457	0.635948
42	6	0	-1.725002	3.787123	-0.536981
43	6	0	-1.013861	5.277257	1.261580
44	1	0	-0.444597	5.453563	2.167808
45	6	0	-1.788391	6.274352	0.674737
46	1	0	-1.829580	7.259239	1.130215
47	6	0	-2.523284	6.031987	-0.504424
48	1	0	-3.115583	6.835190	-0.932426
49	6	0	-2.502230	4.786539	-1.126573
50	1	0	-3.062834	4.590189	-2.033955
51	6	0	1.814228	1.264783	-1.822693
52	6	0	3.352622	1.379117	-2.059156
53	6	0	4.193199	1.962746	-0.875771
54	6	0	5.742332	1.761614	-1.087766
55	6	0	6.330123	0.505508	-0.356326
56	6	0	7.753663	0.117554	-0.887556
57	6	0	8.551989	-0.846196	0.061001

58	6	0	9.488516	-0.077806	1.043581
59	6	0	3.849460	3.461604	-0.639111
60	6	0	9.367860	-1.891292	-0.759494
61	9	0	-3.566130	-2.171655	3.135460
62	9	0	-3.816409	-0.130242	2.346969
63	9	0	-3.845448	-1.326017	-0.294709
64	9	0	-6.412789	-2.830173	0.583367
65	9	0	-6.028168	-1.686154	2.407617
66	9	0	-6.389491	-0.796925	-0.961381
67	9	0	-5.538522	0.570334	0.536414
68	9	0	-7.559460	0.484722	2.085360
69	9	0	-8.496694	-1.231894	1.096900
70	9	0	-7.665530	1.566947	-0.821843
71	9	0	-8.891696	-1.001334	-1.563131
72	9	0	-9.830390	0.885512	-2.053707
73	9	0	-10.600890	-0.318027	-0.406953
74	9	0	-4.341871	-4.240253	1.607509
75	9	0	-2.510382	-3.616528	0.604934
76	9	0	-4.328664	-3.869665	-0.541172
77	9	0	-8.539350	2.775626	1.321951
78	9	0	-10.091218	1.290246	1.713122
79	9	0	-10.203464	2.564330	-0.046766
80	8	0	-1.559932	-0.416468	0.858614
81	8	0	-1.044338	-1.885941	2.520320
82	7	0	0.445167	2.653819	2.034161
83	7	0	0.966467	0.316070	1.648439
84	7	0	2.026027	-1.843384	1.963084
85	7	0	0.617701	-1.834110	-0.012763
86	6	0	1.036480	1.514009	2.336295
87	6	0	1.777852	-0.595251	2.301150
88	6	0	2.369634	0.035655	3.463864
89	6	0	1.901931	1.358321	3.488308
90	6	0	3.241815	-0.444589	4.442942
91	1	0	3.597089	-1.468886	4.414819
92	6	0	3.629690	0.441029	5.445103
93	1	0	4.306819	0.105323	6.224795
94	6	0	3.160315	1.770593	5.469344
95	1	0	3.485549	2.431633	6.267093
96	6	0	2.291241	2.248425	4.491980
97	1	0	1.926095	3.269712	4.501280
98	6	0	1.477334	-2.408591	0.907990
99	6	0	0.316283	-2.790726	-0.965802
100	6	0	0.993375	-4.028488	-0.635956
101	6	0	1.724990	-3.787154	0.536971
102	6	0	1.013848	-5.277289	-1.261588
103	1	0	0.444582	-5.453595	-2.167815
104	6	0	1.788379	-6.274383	-0.674746
105	1	0	1.829568	-7.259270	-1.130224
106	6	0	2.523273	-6.032018	0.504414
107	1	0	3.115573	-6.835220	0.932415

108	6	0	2.502220	-4.786569	1.126563
109	1	0	3.062825	-4.590219	2.033944
110	6	0	-1.814243	-1.264815	1.822685
111	6	0	-3.352637	-1.379141	2.059153
112	6	0	-4.193219	-1.962777	0.875776
113	6	0	-5.742350	-1.761619	1.087766
114	6	0	-6.330111	-0.505491	0.356339
115	6	0	-7.753649	-0.117520	0.887560
116	6	0	-8.551946	0.846260	-0.060990
117	6	0	-9.488467	0.077902	-1.043600
118	6	0	-3.849500	-3.461643	0.639134
119	6	0	-9.367815	1.891352	0.759512

Supplementary Table S5: DFT optimised atomic coordinates of SiPc **3**.

Center Number	Atomic Number	Atomic Type	Coordinates (Angstroms) X	Y	Z
1	14	0	0.000003	-0.000005	-0.000002
2	8	0	-0.136601	-0.000388	-1.804385
3	8	0	1.996251	-0.000194	-2.557763
4	7	0	3.367058	0.000435	0.161203
5	7	0	1.373647	1.376057	0.027170
6	7	0	-0.000409	3.374734	-0.000577
7	7	0	-1.373972	1.375702	-0.027700
8	6	0	2.734495	1.155425	0.104670
9	6	0	1.157001	2.738372	0.028377
10	6	0	2.433534	3.426875	0.088544
11	6	0	3.423340	2.432661	0.140713
12	6	0	2.765089	4.783284	0.105939
13	1	0	1.992626	5.543876	0.066429
14	6	0	4.116704	5.112743	0.174423
15	1	0	4.414378	6.157085	0.188369
16	6	0	5.110826	4.114461	0.225619
17	1	0	6.154618	4.409781	0.277868
18	6	0	4.778396	2.761885	0.209868
19	1	0	5.535482	1.986057	0.247426
20	6	0	-1.157659	2.738075	-0.029299
21	6	0	-2.734771	1.154719	-0.105021
22	6	0	-3.423932	2.431774	-0.141384
23	6	0	-2.434366	3.426245	-0.089579
24	6	0	-4.779077	2.760647	-0.210496
25	1	0	-5.535975	1.984623	-0.247766
26	6	0	-5.111838	4.113137	-0.226581
27	1	0	-6.155707	4.408189	-0.278801
28	6	0	-4.117955	5.111677	-0.175752
29	1	0	-4.415888	6.155942	-0.189948
30	6	0	-2.766254	4.782568	-0.107313
31	1	0	-1.993971	5.543358	-0.068082
32	6	0	0.793587	-0.000484	-2.761082
33	6	0	0.179034	-0.001055	-4.174946

34	6	0	-0.696310	1.259957	-4.340565
35	1	0	-1.501994	1.280334	-3.602779
36	1	0	-1.142640	1.277077	-5.341136
37	1	0	-0.101394	2.172718	-4.224765
38	6	0	-0.694763	-1.263214	-4.340076
39	1	0	-1.500309	-1.284397	-3.602160
40	1	0	-0.098684	-2.175193	-4.224104
41	1	0	-1.141216	-1.281172	-5.340574
42	6	0	1.308636	-0.000587	-5.215931
43	1	0	0.887069	-0.001177	-6.227142
44	1	0	1.947242	-0.881040	-5.105690
45	1	0	1.946011	0.880838	-5.106249
46	8	0	0.136601	0.000381	1.804380
47	8	0	-1.996255	0.000191	2.557744
48	7	0	-3.367054	-0.000444	-0.161197
49	7	0	-1.373642	-1.376066	-0.027173
50	7	0	0.000413	-3.374744	0.000572
51	7	0	1.373976	-1.375711	0.027699
52	6	0	-2.734490	-1.155434	-0.104668
53	6	0	-1.156996	-2.738381	-0.028382
54	6	0	-2.433529	-3.426885	-0.088545
55	6	0	-3.423335	-2.432671	-0.140709
56	6	0	-2.765084	-4.783293	-0.105939
57	1	0	-1.992620	-5.543886	-0.066433
58	6	0	-4.116700	-5.112752	-0.174418
59	1	0	-4.414373	-6.157095	-0.188364
60	6	0	-5.110821	-4.114471	-0.225610
61	1	0	-6.154613	-4.409790	-0.277854
62	6	0	-4.778391	-2.761894	-0.209858
63	1	0	-5.535478	-1.986067	-0.247413
64	6	0	1.157664	-2.738084	0.029295
65	6	0	2.734775	-1.154728	0.105025
66	6	0	3.423935	-2.431784	0.141390
67	6	0	2.434370	-3.426254	0.089581
68	6	0	4.779080	-2.760656	0.210508
69	1	0	5.535978	-1.984631	0.247783
70	6	0	5.111842	-4.113145	0.226594
71	1	0	6.155710	-4.408198	0.278818
72	6	0	4.117959	-5.111686	0.175760
73	1	0	4.415892	-6.155951	0.189957
74	6	0	2.766258	-4.782577	0.107316
75	1	0	1.993975	-5.543367	0.068081
76	6	0	-0.793592	0.000475	2.761072
77	6	0	-0.179049	0.001084	4.174940
78	6	0	0.696328	-1.259901	4.340590
79	1	0	1.502019	-1.280269	3.602811
80	1	0	1.142650	-1.276988	5.341165
81	1	0	0.101439	-2.172680	4.224803
82	6	0	0.694714	1.263271	4.340050
83	1	0	1.500265	1.284460	3.602139
84	1	0	0.098611	2.175231	4.224055
85	1	0	1.141160	1.281261	5.340551
86	6	0	-1.308657	0.000607	5.215918
87	1	0	-0.887097	0.001229	6.227131
88	1	0	-1.947286	0.881041	5.105655

89	1	0	-1.946007	-0.880837	5.106250

Supplementary Table S6: DFT optimised atomic coordinates of SiPc **4**.

Center Number	Atomic Number	Atomic Type	Coordinates (Angstroms) X	Y	Z
1	14	0	0.000000	0.000000	0.000000
2	9	0	-3.161348	1.433033	2.236653
3	9	0	-4.839076	2.673159	1.664893
4	9	0	-4.163619	1.082774	0.335541
5	8	0	-1.103078	1.378450	0.419971
6	8	0	-1.525847	2.480025	-1.511200
7	7	0	0.344978	0.738113	-3.269811
8	7	0	1.207804	1.114351	-1.034987
9	7	0	2.685899	1.913567	0.713357
10	7	0	0.971927	0.452844	1.616235
11	6	0	1.186770	1.270346	-2.407399
12	6	0	2.285480	1.821930	-0.540955
13	6	0	2.970185	2.482396	-1.636215
14	6	0	2.282092	2.133003	-2.808187
15	6	0	4.093395	3.311128	-1.672822
16	1	0	4.620407	3.572084	-0.761357
17	6	0	4.503398	3.780711	-2.918323
18	1	0	5.372667	4.427923	-2.988141
19	6	0	3.811249	3.430875	-4.095687
20	1	0	4.161096	3.815953	-5.048963
21	6	0	2.692309	2.602383	-4.057330
22	1	0	2.153284	2.326544	-4.957202
23	6	0	2.074825	1.280520	1.697257
24	6	0	0.651585	0.037904	2.895226
25	6	0	1.596199	0.612429	3.833867
26	6	0	2.491675	1.389199	3.082165
27	6	0	1.717839	0.504162	5.220897
28	1	0	1.022060	-0.100733	5.792238
29	6	0	2.761088	1.197182	5.829665
30	1	0	2.888721	1.136995	6.906466
31	6	0	3.660249	1.978075	5.074392
32	1	0	4.462382	2.503102	5.584623
33	6	0	3.538260	2.085317	3.691403
34	1	0	4.225426	2.681854	3.100976
35	6	0	-1.740725	2.259552	-0.333605
36	6	0	-2.809950	3.047404	0.466927
37	6	0	-3.734614	2.050990	1.184175
38	6	0	-3.649053	3.893020	-0.504742
39	1	0	-4.363554	4.511030	0.043342
40	1	0	-4.195577	3.257770	-1.205463
41	1	0	-2.988142	4.534980	-1.088313
42	6	0	-2.112074	3.938453	1.515448
43	1	0	-1.487329	3.344351	2.183780
44	1	0	-2.856790	4.473998	2.111760
45	1	0	-1.484053	4.677510	1.010242
46	9	0	3.161348	-1.433033	-2.236653

47	9	0	4.839076	-2.673159	-1.664893
48	9	0	4.163619	-1.082774	-0.335541
49	8	0	1.103078	-1.378450	-0.419971
50	8	0	1.525847	-2.480025	1.511200
51	7	0	-0.344978	-0.738113	3.269811
52	7	0	-1.207804	-1.114351	1.034987
53	7	0	-2.685899	-1.913567	-0.713357
54	7	0	-0.971927	-0.452844	-1.616235
55	6	0	-1.186770	-1.270346	2.407399
56	6	0	-2.285480	-1.821930	0.540955
57	6	0	-2.970185	-2.482396	1.636215
58	6	0	-2.282092	-2.133003	2.808187
59	6	0	-4.093395	-3.311128	1.672822
60	1	0	-4.620407	-3.572084	0.761357
61	6	0	-4.503398	-3.780711	2.918323
62	1	0	-5.372667	-4.427923	2.988141
63	6	0	-3.811249	-3.430875	4.095687
64	1	0	-4.161096	-3.815953	5.048963
65	6	0	-2.692309	-2.602383	4.057330
66	1	0	-2.153284	-2.326544	4.957202
67	6	0	-2.074825	-1.280520	-1.697257
68	6	0	-0.651585	-0.037904	-2.895226
69	6	0	-1.596199	-0.612429	-3.833867
70	6	0	-2.491675	-1.389199	-3.082165
71	6	0	-1.717839	-0.504162	-5.220897
72	1	0	-1.022060	0.100733	-5.792238
73	6	0	-2.761088	-1.197182	-5.829665
74	1	0	-2.888721	-1.136995	-6.906466
75	6	0	-3.660249	-1.978075	-5.074392
76	1	0	-4.462382	-2.503102	-5.584623
77	6	0	-3.538260	-2.085317	-3.691403
78	1	0	-4.225426	-2.681854	-3.100976
79	6	0	1.740725	-2.259552	0.333605
80	6	0	2.809950	-3.047404	-0.466927
81	6	0	3.734614	-2.050990	-1.184175
82	6	0	3.649053	-3.893020	0.504742
83	1	0	4.363554	-4.511030	-0.043342
84	1	0	4.195577	-3.257770	1.205463
85	1	0	2.988142	-4.534980	1.088313
86	6	0	2.112074	-3.938453	-1.515448
87	1	0	1.487329	-3.344351	-2.183780
88	1	0	2.856790	-4.473998	-2.111760
89	1	0	1.484053	-4.677510	-1.010242

Supplementary Table S7: DFT optimised atomic coordinates of SiPc **5**.

Center Number	Atomic Number	Atomic Type	Coordinates (Angstroms) X	Y	Z
1	14	0	-0.000197	0.000665	-0.000202
2	8	0	1.797776	0.040053	-0.208466
3	8	0	2.264354	2.152771	-0.866427
4	7	0	0.579532	-3.229264	0.770694

5	7	0	0.044438	-1.663976	-1.003322
6	7	0	-0.380697	-0.841941	-3.245535
7	7	0	-0.352775	0.978848	-1.643518
8	6	0	0.315114	-2.913433	-0.481155
9	6	0	-0.136529	-1.795575	-2.364606
10	6	0	0.003552	-3.192294	-2.734025
11	6	0	0.291570	-3.892977	-1.552162
12	6	0	-0.091184	-3.848520	-3.962967
13	1	0	-0.313029	-3.297034	-4.870387
14	6	0	0.108780	-5.226918	-3.971573
15	1	0	0.041880	-5.773062	-4.907897
16	6	0	0.396638	-5.931104	-2.784631
17	1	0	0.545486	-7.005911	-2.830261
18	6	0	0.491979	-5.274714	-1.559734
19	1	0	0.711161	-5.807427	-0.640686
20	6	0	-0.480306	0.429966	-2.902267
21	6	0	-0.573885	2.338157	-1.740972
22	6	0	-0.837333	2.680298	-3.126698
23	6	0	-0.774662	1.483089	-3.857071
24	6	0	-1.110380	3.897813	-3.753092
25	1	0	-1.153649	4.817813	-3.180149
26	6	0	-1.319271	3.879307	-5.130194
27	1	0	-1.532941	4.807026	-5.652807
28	6	0	-1.257914	2.676682	-5.863257
29	1	0	-1.425862	2.701578	-6.935924
30	6	0	-0.985467	1.462527	-5.237340
31	1	0	-0.935960	0.531785	-5.792364
32	6	0	2.621022	1.021968	-0.573029
33	6	0	4.104002	0.629882	-0.536283
34	6	0	4.673017	1.112138	0.831171
35	1	0	4.487986	2.187615	0.938800
36	1	0	4.141748	0.606616	1.648077
37	6	0	6.182866	0.813181	0.912990
38	1	0	6.565242	1.158299	1.882486
39	6	0	6.915368	1.550220	-0.227152
40	1	0	7.995356	1.361933	-0.164181
41	1	0	6.772840	2.634256	-0.127725
42	6	0	6.369337	1.067548	-1.587175
43	1	0	6.886431	1.595148	-2.399010
44	6	0	4.860006	1.367006	-1.669340
45	1	0	4.667608	2.441272	-1.591368
46	1	0	4.464544	1.045527	-2.642006
47	6	0	4.352510	-0.890824	-0.665802
48	1	0	3.819587	-1.424309	0.127370
49	1	0	3.946099	-1.253701	-1.618617
50	6	0	5.865434	-1.187605	-0.586178
51	1	0	6.023928	-2.269403	-0.683278
52	6	0	6.413052	-0.705929	0.773776
53	1	0	7.484459	-0.933688	0.851905
54	1	0	5.912452	-1.240033	1.592217
55	6	0	6.601849	-0.451428	-1.723928
56	1	0	7.676296	-0.675731	-1.685972
57	1	0	6.237117	-0.802744	-2.698312
58	8	0	-1.798555	-0.038610	0.208066
59	8	0	-2.264804	-2.130620	0.860004

60	7	0	-0.580012	3.230706	-0.771111
61	7	0	-0.045024	1.665293	1.003006
62	7	0	0.379945	0.842998	3.245236
63	7	0	0.352168	-0.977562	1.643085
64	6	0	-0.315659	2.914850	0.480901
65	6	0	0.135835	1.796757	2.364356
66	6	0	-0.004252	3.193435	2.733857
67	6	0	-0.292162	3.894276	1.551996
68	6	0	0.090394	3.849563	3.962886
69	1	0	0.312145	3.297990	4.870276
70	6	0	-0.109532	5.227952	3.971601
71	1	0	-0.042698	5.774000	4.907986
72	6	0	-0.397277	5.932288	2.784684
73	1	0	-0.546100	7.007095	2.830423
74	6	0	-0.492538	5.276055	1.559714
75	1	0	-0.711636	5.808892	0.640714
76	6	0	0.479586	-0.428824	2.901856
77	6	0	0.573299	-2.336815	1.740437
78	6	0	0.836683	-2.679097	3.126156
79	6	0	0.773915	-1.482020	3.856621
80	6	0	1.109726	-3.896644	3.752402
81	1	0	1.153059	-4.816566	3.179345
82	6	0	1.318529	-3.878300	5.129535
83	1	0	1.532198	-4.806070	5.652057
84	6	0	1.257076	-2.675785	5.862709
85	1	0	1.424949	-2.700785	6.935386
86	6	0	0.984618	-1.461570	5.236880
87	1	0	0.935029	-0.530881	5.791986
88	6	0	-2.618887	-1.017666	0.571534
89	6	0	-4.101897	-0.630310	0.536266
90	6	0	-4.670711	-1.114061	-0.830803
91	1	0	-4.482582	-2.188912	-0.938744
92	1	0	-4.141679	-0.606858	-1.648096
93	6	0	-6.181489	-0.819510	-0.911134
94	1	0	-6.563734	-1.165589	-1.880320
95	6	0	-6.910765	-1.558884	0.229555
96	1	0	-7.991351	-1.373751	0.167610
97	1	0	-6.765147	-2.642471	0.129802
98	6	0	-6.364908	-1.074866	1.589151
99	1	0	-6.879696	-1.604129	2.401348
100	6	0	-4.854666	-1.369904	1.669900
101	1	0	-4.659099	-2.443515	1.591529
102	1	0	-4.459257	-1.047446	2.642217
103	6	0	-4.354655	0.889650	0.666278
104	1	0	-3.824042	1.424842	-0.127276
105	1	0	-3.948434	1.253543	1.618762
106	6	0	-5.868527	1.181938	0.588090
107	1	0	-6.030090	2.263231	0.685528
108	6	0	-6.416002	0.698889	-0.771446
109	1	0	-7.488139	0.923517	-0.848532
110	1	0	-5.917739	1.234603	-1.590261
111	6	0	-6.601750	0.443391	1.726389
112	1	0	-7.676880	0.664554	1.689447
113	1	0	-6.237165	0.795604	2.700498

Supplementary Table S8: DFT optimised atomic coordinates of SiPc **6**.

Center Number	Atomic Number	Atomic Type	Coordinates (Angstroms) X	Y	Z
1	14	0	0.001239	0.002764	-0.002000
2	9	0	7.424865	2.673138	0.623075
3	9	0	6.444204	2.851412	-1.306807
4	9	0	5.331291	3.272061	0.511176
5	9	0	7.546725	-3.027374	-1.110510
6	9	0	7.162795	-3.390265	0.993926
7	9	0	8.698483	-1.963135	0.397559
8	9	0	-7.429492	-2.650750	-0.706451
9	9	0	-6.406384	-2.907144	1.192609
10	9	0	-5.332696	-3.247093	-0.665232
11	9	0	-7.784584	2.722974	1.288213
12	9	0	-7.071784	3.546820	-0.591029
13	9	0	-8.632913	2.024475	-0.586103
14	8	0	1.822422	-0.064765	0.025935
15	8	0	2.344532	-2.272621	0.058353
16	8	0	-1.819646	0.069971	-0.033919
17	8	0	-2.343865	2.277551	-0.031669
18	7	0	-0.059484	-0.003571	3.371513
19	7	0	0.155752	1.359739	1.376134
20	7	0	0.531154	3.331859	0.013891
21	7	0	0.202438	1.365467	-1.368486
22	7	0	0.062804	0.008951	-3.375130
23	7	0	-0.151655	-1.354677	-1.379837
24	7	0	-0.529542	-3.326170	-0.017512
25	7	0	-0.201384	-1.359532	1.364771
26	6	0	0.107959	1.143609	2.740114
27	6	0	0.386253	2.706766	1.164665
28	6	0	0.473818	3.383740	2.444177
29	6	0	0.294525	2.403890	3.433070
30	6	0	0.685214	4.722756	2.779296
31	1	0	0.821817	5.472439	2.007545
32	6	0	0.710835	5.048485	4.133119
33	1	0	0.872017	6.079445	4.433593
34	6	0	0.531290	4.064016	5.126495
35	1	0	0.557424	4.356690	6.171893
36	6	0	0.321690	2.728690	4.791178
37	1	0	0.182921	1.964111	5.547964
38	6	0	0.425380	2.711433	-1.143799
39	6	0	0.204835	1.154225	-2.734071
40	6	0	0.415552	2.417148	-3.415182
41	6	0	0.558400	3.393355	-2.416741
42	6	0	0.492969	2.747026	-4.770149
43	1	0	0.382540	1.985275	-5.534419
44	6	0	0.715045	4.083513	-5.092562

45	1	0	0.780113	4.379946	-6.135193
46	6	0	0.857858	5.064297	-4.089621
47	1	0	1.030363	6.096285	-4.380094
48	6	0	0.782206	4.733544	-2.738893
49	1	0	0.890482	5.480337	-1.959868
50	6	0	-0.102957	-1.138544	-2.743888
51	6	0	-0.382141	-2.701595	-1.168402
52	6	0	-0.467407	-3.378936	-2.447873
53	6	0	-0.287477	-2.399137	-3.436772
54	6	0	-0.677507	-4.718177	-2.782976
55	1	0	-0.814708	-5.467761	-2.011234
56	6	0	-0.701091	-5.044239	-4.136738
57	1	0	-0.861192	-6.075372	-4.437190
58	6	0	-0.520915	-4.059861	-5.130104
59	1	0	-0.545481	-4.352800	-6.175466
60	6	0	-0.312725	-2.724315	-4.794845
61	1	0	-0.173577	-1.959823	-5.551650
62	6	0	-0.425478	-2.705391	1.140007
63	6	0	-0.203822	-1.148455	2.730257
64	6	0	-0.416993	-2.411016	3.411353
65	6	0	-0.560624	-3.387015	2.412899
66	6	0	-0.495736	-2.740635	4.766274
67	1	0	-0.384572	-1.979007	5.530557
68	6	0	-0.720091	-4.076777	5.088640
69	1	0	-0.786286	-4.373041	6.131247
70	6	0	-0.863699	-5.057384	4.085674
71	1	0	-1.037928	-6.089100	4.376087
72	6	0	-0.786584	-4.726835	2.734955
73	1	0	-0.895346	-5.473537	1.955912
74	6	0	2.659195	-1.092543	0.047201
75	6	0	4.105222	-0.671836	0.054192
76	6	0	4.490554	0.672742	0.035416
77	1	0	3.732315	1.445398	0.021310
78	6	0	5.844294	1.007907	0.047301
79	6	0	6.821676	0.010442	0.077801
80	1	0	7.872190	0.274208	0.100052
81	6	0	6.433416	-1.328936	0.097447
82	6	0	5.080728	-1.671293	0.085654
83	1	0	4.761307	-2.707360	0.108650
84	6	0	6.260624	2.453910	-0.026784
85	6	0	7.466055	-2.426315	0.097882
86	6	0	-2.657487	1.097163	-0.038488
87	6	0	-4.102991	0.674978	-0.050328
88	6	0	-4.485881	-0.669166	-0.075844
89	1	0	-3.726434	-1.440654	-0.091600
90	6	0	-5.840079	-1.006370	-0.095120
91	6	0	-6.817632	-0.010441	-0.086702
92	1	0	-7.868098	-0.274880	-0.117872
93	6	0	-6.431355	1.330891	-0.058959
94	6	0	-5.080462	1.674704	-0.041905

95	1	0	-4.763928	2.711330	-0.031425
96	6	0	-6.252114	-2.455408	-0.073160
97	6	0	-7.481681	2.409322	0.007896

Supplementary Table S9: DFT optimised atomic coordinates of SiPc **7**.

Center Number	Atomic Number	Atomic Type	Coordinates (Angstroms) X	Y	Z
1	14	0	0.000002	0.000023	0.000015
2	8	0	-1.807395	-0.018048	-0.000754
3	8	0	-2.375338	-2.212945	-0.003208
4	8	0	-8.092754	0.631022	-0.002048
5	7	0	-0.001643	-0.002499	3.374172
6	7	0	-0.144867	1.365660	1.376528
7	7	0	-0.447965	3.341266	0.002299
8	7	0	-0.143526	1.367719	-1.374588
9	6	0	-0.126749	1.148652	2.738592
10	6	0	-0.336741	2.714932	1.156986
11	6	0	-0.428415	3.397786	2.434632
12	6	0	-0.292174	2.415381	3.427991
13	6	0	-0.608743	4.742255	2.764878
14	1	0	-0.710546	5.493863	1.989458
15	6	0	-0.648556	5.071867	4.117675
16	1	0	-0.786143	6.107706	4.413676
17	6	0	-0.512965	4.084922	5.115168
18	1	0	-0.548810	4.379838	6.159796
19	6	0	-0.333873	2.743748	4.784527
20	1	0	-0.228604	1.976685	5.544314
21	6	0	-0.335544	2.716677	-1.153216
22	6	0	-0.124054	1.152748	-2.736964
23	6	0	-0.288712	2.420520	-3.424624
24	6	0	-0.425867	3.401451	-2.429933
25	6	0	-0.329033	2.750922	-4.780712
26	1	0	-0.223059	1.984987	-5.541538
27	6	0	-0.507690	4.092603	-5.109519
28	1	0	-0.542466	4.389085	-6.153741
29	6	0	-0.644201	5.078068	-4.110688
30	1	0	-0.781416	6.114359	-4.405281
31	6	0	-0.605757	4.746430	-2.758348
32	1	0	-0.708271	5.496882	-1.981903
33	6	0	-2.680425	-1.027621	-0.002072
34	6	0	-4.103781	-0.574967	-0.002051
35	6	0	-4.471325	0.781016	-0.001334
36	1	0	-3.697022	1.539488	-0.000708
37	6	0	-5.808334	1.149715	-0.001358
38	1	0	-6.107724	2.192780	-0.000840
39	6	0	-6.812546	0.167094	-0.002072

40	6	0	-6.458057	-1.190078	-0.002794
41	1	0	-7.215497	-1.964758	-0.003346
42	6	0	-5.110889	-1.545569	-0.002803
43	1	0	-4.817213	-2.589985	-0.003411
44	6	0	-9.167340	-0.308272	-0.001993
45	1	0	-9.101555	-0.953804	0.885886
46	1	0	-9.102167	-0.953225	-0.890342
47	6	0	-10.472362	0.475967	-0.001307
48	1	0	-10.488213	1.131295	-0.880805
49	1	0	-10.487473	1.130965	0.878446
50	6	0	-11.704505	-0.437533	-0.000986
51	1	0	-11.672213	-1.097946	-0.879206
52	1	0	-11.671580	-1.098191	0.877027
53	6	0	-13.029427	0.335968	-0.000398
54	1	0	-13.063046	0.996920	-0.877940
55	1	0	-13.062550	0.996438	0.877522
56	6	0	-14.265646	-0.571968	-0.000293
57	1	0	-14.230076	-1.233364	0.877272
58	1	0	-14.230529	-1.232953	-0.878186
59	6	0	-15.592078	0.198283	0.000238
60	1	0	-15.628266	0.859401	0.878085
61	1	0	-15.628698	0.859864	-0.877245
62	6	0	-16.828648	-0.709550	0.000308
63	1	0	-16.791878	-1.370177	0.877326
64	1	0	-16.792277	-1.369763	-0.877038
65	6	0	-18.149364	0.066895	0.000799
66	1	0	-18.231189	0.710239	-0.882818
67	1	0	-18.230662	0.710005	0.884637
68	1	0	-19.011462	-0.608203	0.000967
69	8	0	1.807400	0.018088	0.000786
70	8	0	2.375369	2.212980	0.003192
71	8	0	8.092750	-0.631055	0.002009
72	7	0	0.001641	0.002545	-3.374142
73	7	0	0.144866	-1.365614	-1.376497
74	7	0	0.447951	-3.341221	-0.002266
75	7	0	0.143527	-1.367672	1.374620
76	6	0	0.126746	-1.148607	-2.738562
77	6	0	0.336732	-2.714888	-1.156954
78	6	0	0.428406	-3.397743	-2.434601
79	6	0	0.292169	-2.415337	-3.427961
80	6	0	0.608730	-4.742213	-2.764846
81	1	0	0.710530	-5.493822	-1.989426
82	6	0	0.648544	-5.071826	-4.117643
83	1	0	0.786128	-6.107665	-4.413644
84	6	0	0.512958	-4.084881	-5.115137
85	1	0	0.548803	-4.379798	-6.159765
86	6	0	0.333869	-2.743705	-4.784499
87	1	0	0.228604	-1.976643	-5.544286
88	6	0	0.335536	-2.716630	1.153249
89	6	0	0.124052	-1.152701	2.736995

90	6	0	0.288708	-2.420472	3.424655
91	6	0	0.425859	-3.401403	2.429966
92	6	0	0.329027	-2.750871	4.780743
93	1	0	0.223055	-1.984936	5.541567
94	6	0	0.507680	-4.092552	5.109552
95	1	0	0.542455	-4.389033	6.153773
96	6	0	0.644187	-5.078020	4.110722
97	1	0	0.781398	-6.114310	4.405316
98	6	0	0.605744	-4.746383	2.758382
99	1	0	0.708255	-5.496837	1.981938
100	6	0	2.680441	1.027652	0.002098
101	6	0	4.103791	0.574982	0.002050
102	6	0	4.471319	-0.781006	0.001351
103	1	0	3.697007	-1.539469	0.000757
104	6	0	5.808323	-1.149721	0.001360
105	1	0	6.107701	-2.192789	0.000860
106	6	0	6.812547	-0.167111	0.002036
107	6	0	6.458074	1.190065	0.002737
108	1	0	7.215524	1.964736	0.003265
109	6	0	5.110911	1.545572	0.002763
110	1	0	4.817247	2.589992	0.003358
111	6	0	9.167348	0.308225	0.001916
112	1	0	9.101569	0.953729	-0.885983
113	1	0	9.102186	0.953208	0.890245
114	6	0	10.472360	-0.476031	0.001253
115	1	0	10.488203	-1.131329	0.880774
116	1	0	10.487461	-1.131059	-0.878477
117	6	0	11.704515	0.437453	0.000901
118	1	0	11.672229	1.097900	0.879097
119	1	0	11.671601	1.098079	-0.877136
120	6	0	13.029427	-0.336064	0.000348
121	1	0	13.063033	-0.996984	0.877915
122	1	0	13.062546	-0.996568	-0.877547
123	6	0	14.265657	0.571856	0.000216
124	1	0	14.230103	1.233217	-0.877377
125	1	0	14.230541	1.232877	0.878082
126	6	0	15.592079	-0.198412	-0.000272
127	1	0	15.628268	-0.859566	-0.878092
128	1	0	15.628683	-0.859957	0.877239
129	6	0	16.828661	0.709406	-0.000367
130	1	0	16.791908	1.369997	-0.877413
131	1	0	16.792288	1.369656	0.876951
132	6	0	18.149367	-0.067055	-0.000811
133	1	0	18.231175	-0.710363	0.882834
134	1	0	18.230666	-0.710204	-0.884621
135	1	0	19.011474	0.608032	-0.000998

Supplementary Table S10: DFT optimised atomic coordinates of SiPc **R1**.

Center Number	Atomic Number	Atomic Type	Coordinates (Angstroms) X	Y	Z
1	14	0	-0.019013	0.121431	0.049516
2	8	0	0.369149	0.400529	-1.693096
3	8	0	0.102345	-1.612077	-2.701794
4	7	0	0.676223	3.409668	0.303816
5	7	0	1.579045	1.162160	0.435684
6	7	0	3.224189	-0.609024	0.632653
7	7	0	1.026471	-1.515648	0.150239
8	6	0	1.650315	2.540077	0.483416
9	6	0	2.846207	0.657285	0.638066
10	6	0	3.772561	1.755696	0.846867
11	6	0	3.021602	2.937642	0.745696
12	6	0	5.146772	1.787817	1.095527
13	1	0	5.718235	0.869228	1.174160
14	6	0	5.744229	3.038211	1.239104
15	1	0	6.810307	3.103916	1.435403
16	6	0	4.989644	4.224859	1.138325
17	1	0	5.489945	5.181369	1.257314
18	6	0	3.619280	4.191055	0.890981
19	1	0	3.029337	5.097944	0.813375
20	6	0	2.377821	-1.600107	0.413308
21	6	0	0.530561	-2.797866	0.022391
22	6	0	1.614288	-3.747750	0.196734
23	6	0	2.775236	-2.996068	0.437745
24	6	0	1.649634	-5.142908	0.157795
25	1	0	0.747690	-5.714601	-0.032303
26	6	0	2.880210	-5.760566	0.368687
27	1	0	2.947486	-6.844235	0.345039
28	6	0	4.045504	-5.005574	0.612503
29	1	0	4.987233	-5.522417	0.771973
30	6	0	4.009315	-3.613708	0.650974
31	1	0	4.900398	-3.023838	0.837502
32	6	0	0.402699	-0.431370	-2.737334
33	6	0	0.854685	0.271151	-4.012016
34	1	0	0.061458	0.973233	-4.299198
35	1	0	0.921722	-0.492072	-4.792539
36	6	0	2.180568	1.036963	-3.864501
37	1	0	2.391001	1.558491	-4.807436
38	1	0	2.055956	1.810671	-3.098601
39	6	0	3.369967	0.139785	-3.499353
40	1	0	3.508576	-0.621098	-4.280524
41	1	0	3.139410	-0.412961	-2.578766
42	6	0	4.678808	0.913544	-3.297207
43	1	0	4.523374	1.693168	-2.538173
44	1	0	4.940886	1.441525	-4.225519

45	6	0	5.849967	0.020793	-2.867671
46	1	0	5.574375	-0.510153	-1.945376
47	1	0	6.013474	-0.756472	-3.628038
48	6	0	7.157715	0.788774	-2.637162
49	1	0	6.994055	1.557140	-1.867907
50	1	0	7.428964	1.331202	-3.554340
51	6	0	8.327265	-0.110352	-2.216467
52	1	0	8.055178	-0.653311	-1.299619
53	1	0	8.486916	-0.880390	-2.984881
54	6	0	9.639270	0.649604	-1.983296
55	1	0	9.481427	1.420673	-1.215298
56	1	0	9.913378	1.190329	-2.900495
57	6	0	10.804042	-0.255006	-1.561184
58	1	0	10.959649	-1.026570	-2.328883
59	1	0	10.529516	-0.794954	-0.643449
60	6	0	12.119371	0.499571	-1.329347
61	1	0	11.964900	1.271824	-0.561978
62	1	0	12.395525	1.037965	-2.247324
63	6	0	13.280406	-0.409459	-0.906474
64	1	0	13.003880	-0.947010	0.011979
65	1	0	13.433038	-1.182467	-1.673341
66	6	0	14.598341	0.340908	-0.675832
67	1	0	14.446511	1.114289	0.090859
68	1	0	14.875914	0.877536	-1.594436
69	6	0	15.756738	-0.571283	-0.252566
70	1	0	15.478978	-1.107216	0.666430
71	1	0	15.907061	-1.345332	-1.018851
72	6	0	17.076631	0.175957	-0.023021
73	1	0	16.927001	0.949961	0.743455
74	1	0	17.354828	0.711631	-0.942005
75	6	0	18.233403	-0.738482	0.399787
76	1	0	17.955176	-1.273725	1.319025
77	1	0	18.381935	-1.512906	-0.366467
78	6	0	19.554651	0.006620	0.628431
79	1	0	19.406730	0.780558	1.395341
80	1	0	19.832673	0.542318	-0.290630
81	6	0	20.710849	-0.908946	1.049700
82	1	0	20.859256	-1.683166	0.283015
83	1	0	20.433939	-1.444885	1.969089
84	6	0	22.033042	-0.165355	1.277999
85	1	0	22.309272	0.370646	0.359629
86	1	0	21.885689	0.606815	2.045507
87	6	0	23.182347	-1.088184	1.695835
88	1	0	23.377790	-1.848714	0.931061
89	1	0	22.949818	-1.613221	2.629523
90	1	0	24.110388	-0.528327	1.852021
91	8	0	-0.405232	-0.158785	1.792350
92	8	0	-0.147191	1.854447	2.801804
93	7	0	-0.714762	-3.166941	-0.201924
94	7	0	-1.616901	-0.919267	-0.336583

95	7	0	-3.261226	0.851997	-0.538266
96	7	0	-1.064364	1.758205	-0.051045
97	6	0	-1.688144	-2.297215	-0.384383
98	6	0	-2.883100	-0.414268	-0.543865
99	6	0	-3.808682	-1.512515	-0.756955
100	6	0	-3.058448	-2.694599	-0.652064
101	6	0	-5.181559	-1.544383	-1.012699
102	1	0	-5.752482	-0.625702	-1.094219
103	6	0	-5.778506	-2.794726	-1.159489
104	1	0	-6.843507	-2.860220	-1.361642
105	6	0	-5.024687	-3.981535	-1.054556
106	1	0	-5.524558	-4.937948	-1.176045
107	6	0	-3.655601	-3.947938	-0.800234
108	1	0	-3.066192	-4.854912	-0.719677
109	6	0	-2.415521	1.842730	-0.315171
110	6	0	-0.569039	3.040406	0.079833
111	6	0	-1.653159	3.990186	-0.092660
112	6	0	-2.813528	3.238565	-0.336446
113	6	0	-1.689150	5.385219	-0.050436
114	1	0	-0.787614	5.956864	0.141719
115	6	0	-2.919867	6.002812	-0.260751
116	1	0	-2.987678	7.086388	-0.234491
117	6	0	-4.084610	5.247871	-0.507310
118	1	0	-5.026458	5.764663	-0.666243
119	6	0	-4.047725	3.856114	-0.549143
120	1	0	-4.938350	3.266227	-0.737770
121	6	0	-0.438379	0.671462	2.837781
122	6	0	-0.871584	-0.036625	4.116239
123	1	0	-0.054852	-0.709133	4.408913
124	1	0	-0.963005	0.729341	4.891643
125	6	0	-2.170165	-0.848243	3.975157
126	1	0	-2.357007	-1.377883	4.918538
127	1	0	-2.022571	-1.616188	3.207652
128	6	0	-3.393339	0.006032	3.618141
129	1	0	-3.562045	0.752527	4.407253
130	1	0	-3.184463	0.576807	2.703287
131	6	0	-4.669794	-0.817492	3.405894
132	1	0	-4.485037	-1.573072	2.629286
133	1	0	-4.904096	-1.376561	4.323361
134	6	0	-5.881162	0.032264	3.001040
135	1	0	-5.626754	0.611584	2.102026
136	1	0	-6.088994	0.770300	3.789086
137	6	0	-7.145472	-0.792187	2.727116
138	1	0	-6.935341	-1.515462	1.926273
139	1	0	-7.393784	-1.388636	3.616816
140	6	0	-8.358778	0.060493	2.334223
141	1	0	-8.100249	0.677028	1.461017
142	1	0	-8.584074	0.767859	3.145138
143	6	0	-9.613505	-0.762797	2.015856
144	1	0	-9.387548	-1.465880	1.201046

145	1	0	-9.872239	-1.383623	2.885544
146	6	0	-10.825778	0.091967	1.624431
147	1	0	-11.063796	0.782097	2.446454
148	1	0	-10.559982	0.726124	0.766337
149	6	0	-12.072577	-0.731299	1.276162
150	1	0	-11.833526	-1.419127	0.452273
151	1	0	-12.339234	-1.367621	2.132170
152	6	0	-13.283612	0.125074	0.884483
153	1	0	-13.012182	0.769793	0.036183
154	1	0	-13.530682	0.804731	1.712578
155	6	0	-14.525023	-0.697743	0.516448
156	1	0	-14.276941	-1.376384	-0.312284
157	1	0	-14.797493	-1.343383	1.363621
158	6	0	-15.734802	0.159685	0.123257
159	1	0	-15.459277	0.810361	-0.719117
160	1	0	-15.987744	0.833447	0.954418
161	6	0	-16.972948	-0.662438	-0.257115
162	1	0	-16.719286	-1.335878	-1.088368
163	1	0	-17.249467	-1.313348	0.584708
164	6	0	-18.181527	0.195893	-0.651958
165	1	0	-17.903130	0.849623	-1.490997
166	1	0	-18.437873	0.866619	0.180624
167	6	0	-19.418000	-0.625373	-1.039524
168	1	0	-19.161199	-1.296121	-1.872015
169	1	0	-19.697185	-1.279030	-0.200673
170	6	0	-20.625577	0.233441	-1.435682
171	1	0	-20.884662	0.903034	-0.602854
172	1	0	-20.346320	0.888695	-2.273423
173	6	0	-21.861687	-0.586631	-1.827109
174	1	0	-22.141486	-1.240601	-0.989883
175	1	0	-21.602628	-1.255382	-2.659367
176	6	0	-23.061918	0.279893	-2.221902
177	1	0	-23.366449	0.933959	-1.396769
178	1	0	-22.822909	0.920828	-3.078380
179	1	0	-23.926924	-0.332766	-2.496715

Supplementary Table S11: DFT optimised atomic coordinates of SiPc **R2**.

Center Number	Atomic Number	Atomic Type	Coordinates (Angstroms) X	Y	Z
1	14	0	-0.000004	-0.000021	0.000002
2	8	0	1.805625	0.074897	-0.000289
3	8	0	2.310502	2.283149	-0.000111
4	7	0	-0.559341	3.324177	0.000181
5	7	0	-0.188431	1.361198	-1.375438
6	7	0	-0.000502	0.000078	-3.374219
7	7	0	0.188054	-1.361149	-1.375570

8	6	0	-0.427017	2.702928	-1.155002
9	6	0	-0.164430	1.145815	-2.737846
10	6	0	-0.374950	2.406332	-3.426218
11	6	0	-0.544350	3.382822	-2.432084
12	6	0	-0.430603	2.733722	-4.782507
13	1	0	-0.299349	1.971372	-5.542986
14	6	0	-0.657937	4.067866	-5.112043
15	1	0	-0.705896	4.361838	-6.156428
16	6	0	-0.826776	5.048915	-4.113778
17	1	0	-1.001364	6.079374	-4.408967
18	6	0	-0.772855	4.720302	-2.761197
19	1	0	-0.899502	5.467529	-1.985207
20	6	0	0.163616	-1.145696	-2.737956
21	6	0	0.426678	-2.702900	-1.155280
22	6	0	0.543562	-3.382730	-2.432438
23	6	0	0.373863	-2.406183	-3.426464
24	6	0	0.771871	-4.720209	-2.761692
25	1	0	0.898756	-5.467474	-1.985778
26	6	0	0.825298	-5.048760	-4.114308
27	1	0	0.999723	-6.079214	-4.409608
28	6	0	0.656158	-4.067655	-5.112465
29	1	0	0.703733	-4.361578	-6.156882
30	6	0	0.429008	-2.733513	-4.782787
31	1	0	0.297532	-1.971123	-5.543188
32	6	0	2.648885	1.107667	-0.000235
33	6	0	4.091361	0.694221	-0.000315
34	6	0	5.059260	1.704180	-0.000409
35	1	0	4.707105	2.727759	-0.000435
36	6	0	6.421187	1.390560	-0.000457
37	6	0	6.773318	0.031392	-0.000381
38	1	0	7.827894	-0.228938	-0.000384
39	6	0	5.828329	-1.006207	-0.000297
40	6	0	4.474754	-0.650892	-0.000268
41	1	0	3.697844	-1.402944	-0.000207
42	6	0	7.522416	2.468275	-0.000469
43	6	0	8.403027	2.307650	-1.262509
44	1	0	7.806069	2.425338	-2.172918
45	1	0	8.883009	1.325149	-1.303357
46	1	0	9.194705	3.065592	-1.274995
47	6	0	8.402378	2.308159	1.262097
48	1	0	9.194080	3.066077	1.274697
49	1	0	8.882271	1.325638	1.303565
50	1	0	7.804938	2.426230	2.172138
51	6	0	6.939675	3.894315	-0.000935
52	1	0	6.325269	4.082535	-0.887115
53	1	0	7.754796	4.625566	-0.000972
54	1	0	6.324955	4.083004	0.884926
55	6	0	6.300261	-2.473603	-0.000205
56	6	0	7.153895	-2.742309	1.262055
57	1	0	7.503095	-3.781316	1.273244

58	1	0	6.570025	-2.570674	2.172361
59	1	0	8.035530	-2.095742	1.304937
60	6	0	7.153872	-2.742500	-1.262437
61	1	0	6.569979	-2.571031	-2.172761
62	1	0	7.503094	-3.781500	-1.273459
63	1	0	8.035495	-2.095923	-1.305450
64	6	0	5.121301	-3.465054	-0.000119
65	1	0	5.503632	-4.491192	-0.000068
66	1	0	4.489381	-3.347589	-0.886197
67	1	0	4.489429	-3.347487	0.885980
68	8	0	-1.805636	-0.074929	0.000294
69	8	0	-2.310555	-2.283175	0.000124
70	7	0	0.559360	-3.324216	-0.000176
71	7	0	0.188429	-1.361240	1.375442
72	7	0	0.000506	-0.000120	3.374222
73	7	0	-0.188055	1.361106	1.375573
74	6	0	0.427028	-2.702967	1.155006
75	6	0	0.164433	-1.145856	2.737849
76	6	0	0.374957	-2.406373	3.426221
77	6	0	0.544362	-3.382861	2.432087
78	6	0	0.430610	-2.733761	4.782510
79	1	0	0.299354	-1.971412	5.542989
80	6	0	0.657951	-4.067905	5.112046
81	1	0	0.705911	-4.361876	6.156432
82	6	0	0.826795	-5.048952	4.113782
83	1	0	1.001387	-6.079411	4.408971
84	6	0	0.772873	-4.720340	2.761201
85	1	0	0.899524	-5.467566	1.985211
86	6	0	-0.163613	1.145653	2.737959
87	6	0	-0.426666	2.702860	1.155285
88	6	0	-0.543548	3.382690	2.432442
89	6	0	-0.373854	2.406141	3.426467
90	6	0	-0.771849	4.720169	2.761697
91	1	0	-0.898730	5.467435	1.985784
92	6	0	-0.825273	5.048720	4.114313
93	1	0	-0.999693	6.079175	4.409614
94	6	0	-0.656138	4.067613	5.112469
95	1	0	-0.703711	4.361536	6.156886
96	6	0	-0.428996	2.733471	4.782790
97	1	0	-0.297522	1.971080	5.543191
98	6	0	-2.648913	-1.107685	0.000239
99	6	0	-4.091380	-0.694211	0.000317
100	6	0	-5.059300	-1.704151	0.000417
101	1	0	-4.707166	-2.727736	0.000449
102	6	0	-6.421221	-1.390504	0.000461
103	6	0	-6.773324	-0.031328	0.000378
104	1	0	-7.827895	0.229023	0.000375
105	6	0	-5.828314	1.006252	0.000290
106	6	0	-4.474746	0.650910	0.000262
107	1	0	-3.697821	1.402946	0.000196

108	6	0	-7.522472	-2.468195	0.000475
109	6	0	-8.403083	-2.307547	1.262512
110	1	0	-7.806131	-2.425244	2.172923
111	1	0	-8.883045	-1.325035	1.303354
112	1	0	-9.194777	-3.065471	1.274998
113	6	0	-8.402427	-2.308066	-1.262094
114	1	0	-9.194146	-3.065966	-1.274693
115	1	0	-8.882297	-1.325534	-1.303569
116	1	0	-7.804987	-2.426155	-2.172133
117	6	0	-6.939761	-3.894248	0.000949
118	1	0	-6.325361	-4.082476	0.887131
119	1	0	-7.754898	-4.625481	0.000989
120	1	0	-6.325044	-4.082955	-0.884910
121	6	0	-6.300216	2.473657	0.000183
122	6	0	-7.153878	2.742354	-1.262060
123	1	0	-7.503065	3.781364	-1.273258
124	1	0	-6.570034	2.570695	-2.172378
125	1	0	-8.035523	2.095797	-1.304909
126	6	0	-7.153788	2.742597	1.262432
127	1	0	-6.569875	2.571133	2.172744
128	1	0	-7.502986	3.781606	1.273443
129	1	0	-8.035426	2.096043	1.305482
130	6	0	-5.121237	3.465084	0.000046
131	1	0	-5.503546	4.491230	-0.000007
132	1	0	-4.489291	3.347618	0.886106
133	1	0	-4.489394	3.347492	-0.886072

References:

1. Zysman-Colman, E. *et al.* Solution-Processable Silicon Phthalocyanines in Electroluminescent and Photovoltaic Devices. *ACS Appl. Mater. Interfaces* **8**, 9247-9253, doi:10.1021/acsami.5b12408 (2016).
